# *SLC22A1-ABCB1* Haplotype Profiles Predict Imatinib Pharmacokinetics in Asian Patients with Chronic Myeloid Leukemia

**DOI:** 10.1371/journal.pone.0051771

**Published:** 2012-12-18

**Authors:** Onkar Singh, Jason Yongsheng Chan, Keegan Lin, Charles Chuah Thuan Heng, Balram Chowbay

**Affiliations:** 1 Laboratory of Clinical Pharmacology, Division of Medical Sciences, Humphrey Oei Institute of Cancer Research, National Cancer Centre Singapore, Singapore, Singapore; 2 Department of Haematology, Singapore General Hospital, Singapore, Singapore; Nanyang Technological University, Singapore

## Abstract

**Objective:**

This study aimed to explore the influence of SLC22A1, PXR, ABCG2, ABCB1 and CYP3A5*3 genetic polymorphisms on imatinib mesylate (IM) pharmacokinetics in Asian patients with chronic myeloid leukemia (CML).

**Patients and Methods:**

Healthy subjects belonging to three Asian populations (Chinese, Malay, Indian; n = 70 each) and CML patients (n = 38) were enrolled in a prospective pharmacogenetics study. Imatinib trough (C_0h_) and clearance (CL) were determined in the patients at steady state. Haplowalk method was applied to infer the haplotypes and generalized linear model (GLM) to estimate haplotypic effects on IM pharmacokinetics. Association of haplotype copy numbers with IM pharmacokinetics was defined by Mann-Whitney U test.

**Results:**

Global haplotype score statistics revealed a *SLC22A1* sub-haplotypic region encompassing three polymorphisms (rs3798168, rs628031 and IVS7+850C>T), to be significantly associated with IM clearance (*p* = 0.013). Haplotype-specific GLM estimated that the haplotypes AGT and CGC were both associated with 22% decrease in clearance compared to CAC [CL (*10^−2^ L/hr/mg): CAC vs AGT: 4.03 vs 3.16, *p* = 0.017; CAC vs CGC: 4.03 vs 3.15, *p* = 0.017]. Patients harboring 2 copies of AGT or CGC haplotypes had 33.4% lower clearance and 50% higher C_0h_ than patients carrying 0 or 1 copy [CL (*10^−2^ L/hr/mg): 2.19 vs 3.29, *p* = 0.026; C_0h_ (*10^−6^ 1/ml): 4.76 vs 3.17, *p* = 0.013, respectively]. Further subgroup analysis revealed *SLC22A1* and *ABCB1* haplotypic combinations to be significantly associated with clearance and C_0h_ (*p* = 0.002 and 0.009, respectively).

**Conclusion:**

This exploratory study suggests that *SLC22A1-ABCB1* haplotypes may influence IM pharmacokinetics in Asian CML patients.

## Introduction

Chronic myeloid leukemia (CML) is a hematopoietic stem cell disorder, characterized by the presence of the Philadelphia chromosome (Ph) that results from a balanced reciprocal translocation between chromosomes 9 and 22. Functionally, this translocation results in the formation of the *BCR-ABL* gene which is then translated to the BCR-ABL protein with intrinsic tyrosine kinase activity that is critical to the development of CML [1]. Imatinib mesylate (IM), a selective inhibitor of the BCR-ABL tyrosine kinase, has been established as the first-line treatment option for CML since the remarkable success of the IRIS (International Randomized Study of Interferon vs. STI571) trial [2], which demonstrated the dramatic superiority of IM over interferon plus cytarabine - at 18 months, the rate of complete cytogenetic response (CCyR) in patients treated with IM was a remarkable 76% versus 15% for patients treated with interferon plus cytarabine [2].

However, despite significant progress in CML treatment, about 30–40% of the patients still fail to achieve major molecular response (MMR) at 18 months [3]. This is clinically important, since it is now recognized that there is an intimate link between early molecular response and long-term clinical outcome. Recently, an expanded 7 year follow-up study of the IRIS trial found that patients who achieved MMR by 18 months enjoyed remarkably durable responses, with no disease progression and 95% event-free survival at 7 years. The probability of loss of CCyR by 7 years was only 3% for patients in MMR at 18 months compared to 26% for patients with CCyR but not MMR [4]. In the words of the IRIS investigators, achieving an MMR may be a “*safe haven that promises*” favorable long-term outcomes in patients with CML [4].

Suboptimal response and eventual treatment failure may be associated with several factors including the presence of *BCR-ABL* mutations [5] as well as those associated with suboptimal therapeutic drug levels. These include poor medication compliance [6], drug-drug interactions [7], variable metabolizing enzyme activities [8], as well as different efflux and influx transporter activities [9]. Notably, higher plasma IM trough levels have recently been correlated with achievement of CCyR and MMR in Caucasians, with an optimum threshold approximately above 1000 ng/mL [3], [10]. These results have been replicated in studies on the Chinese [11], Japanese [12], Koreans [13], Israelis [14] and Jordanians [15]. However, there are some other studies which did not agree with this finding [16]–[18]. Although there may be good efficacy in escalating the dose of IM above the “standard” 400 mg in clinical practice so as to achieve therapeutic drug levels *in vivo* [19], the high inter-ethnic and inter-individual variability in IM plasma trough levels [3], [10], together with the fact that a significant proportion of patients with IM levels above 1000 ng/mL still do not achieve optimal response, suggest that there are other critical factors that limit IM efficacy.

One plausible hypothesis to explain the wide variability may be a result of disparities in population pharmacokinetics and pharmacogenetics. Differences in activity, expression levels as well as functional single nucleotide polymorphisms (SNPs) of both efflux transporters (ATP-binding cassette transporters, such as ABCB1 and ABCG2) and uptake transporters (solute carriers, such as hOCT-1 and OATP1A2) [20] of IM into leukemic cells have been suggested. Particularly, White *et al.* showed that in patients on IM doses less than 600 mg/day, 82% of those with low activity of hOCT-1, the major active influx pump for IM, failed to achieve MMR by 18 months, compared to only 17% of those with high hOCT-1 activity. Furthermore, the negative impact of low hOCT-1 activity may be overcome by escalating to high dose IM at 800 mg/day [9]. On the other hand, several studies focusing on the impact of *SLC22A1* (encoding for hOCT-1) expression or polymorphisms on IM pharmacokinetics have revealed rather heterogeneous results. High expression levels have been associated with favorable response in some studies [6], [21], [22] but not others [9], [23]. An association between *SLC22A1* SNPs and response to IM has also been observed in some reports [12], [24]–[26], whereas other studies failed to support such findings [27], [28].

To address these issues, we investigated if differences in SNP frequencies of efflux and influx transporters, as well as drug metabolizing enzymes, would be able to account for inter-ethnic and inter-individual variation in IM trough levels. In this exploratory study, the effects of 89 *SLC22A1* SNPs identified on whole gene sequencing, as well as 11 candidate SNPs from *ABCB1*, *ABCG2, CYP3A5* and *PXR* genes on IM pharmacokinetics were examined in a cohort of healthy subjects belonging to Asian populations from Singapore, consisting of Chinese, Malay and Indian ethnic groups as well as patients with Ph+ chronic phase CML.

## Patients, Materials, and Methods

### Ethics Statement

All participants provided written informed consent for the study, which was approved by the ethics review committee of the National Cancer Centre, Singapore.

### Patients and Healthy Participants

A total of 210 healthy subjects belonging to Asian populations from Singapore, consisting of Chinese, Malay and Indian ethnic groups (n = 70 per group) were enrolled for this study. In addition, a total of 38 patients with Ph+ chronic phase CML (32 Chinese, 4 Malays, 2 Indians) who received IM at the Singapore General Hospital from January 2001 to September 2007 were prospectively recruited. Ethnicity of the subjects was confirmed by careful screening and verified against their National Registry Identification Cards. Prior to commencing therapy, all patients were required to have a complete blood count including the white cell count differential, as well as standard biochemistry. Baseline investigations also included bone marrow evaluation for morphology, conventional cytogenetic analysis by G-banding, and *BCR-ABL* fluorescence *in situ* hybridization (FISH) studies. At least 20 metaphases were assessed for cytogenetic analysis. Peripheral blood *BCR-ABL*/total *ABL* ratio was obtained by using quantitative real-time reverse transcription-polymerase chain reaction (RT-PCR). Patient characteristics are summarized in Table 1.

**Table 1:** Demographic and clinicopathological information of patients with CML.

Characteristics	n (%)
**Total**	38 (100)
**Ethnicity**	
Chinese	32 (84.2)
Malay	4 (10.5)
Indian	2 (5.3)
**Gender**	
Male	24 (63.2)
Female	14 (36.8)
**Prior Interferon**	
No	23 (60.5)
Yes	15 (39.5)
**Imatinib dosage (mg)**	
400	32 (84.2)
600	4 (10.5)
300	2 (5.3)
**Treatment response**	
CCyR 6 months	
No	13 (34.2)
Yes	24 (63.2)
Unknown	1 (2.6)
CCyR 12 months	
No	6 (15.8)
Yes	30 (78.9)
Unknown	2 (5.3)
MMR 18 months	
No	14 (36.8)
Yes	14 (36.8)
Unknown	10 (26.3)
Treatment failure	
No	33 (86.8)
Yes	5 (13.2)
**Physical variables**	**Median (range)**
Age (years)	48 (23–79)
Body weight (kg)	67.5 (47–135.5)
Height (cm)	165.5 (145–180)
BMI (kg/m^2^)	24.4 (17.9–49.5)
BSA (m^2^)	1.8 (1.4–2.5)

Abbreviations: CCyR, complete cytogenetic response; MMR, major molecular response; BMI, body mass index; BSA, body surface area.

### Patient Monitoring and Response to Therapy

Patients were monitored regularly for treatment response on an outpatient basis. A complete hematological response was defined as normalization of peripheral blood count and disappearance of splenomegaly, if present, within 3 months. To evaluate for cytogenetic response, a repeat bone marrow biopsy was performed 6 monthly after treatment with IM. Cytogenetic response was defined using standard convention with respect to the percentage of Ph-positive cells in the bone marrow as complete (CCyR, 0%), partial (PCyR, 1–34%) and minor (35–90%). A <1% *BCR-ABL*/total *ABL* ratio obtained from RT-PCR was also taken to indicate CCyR [4]. A major cytogenetic response (MCyR) was defined as the sum of complete and partial cytogenetic responses. The quantification of peripheral blood *BCR-ABL*/total *ABL* ratio was repeated every 3 months after treatment with IM. According to the definition of the international scale [29], major molecular response (MMR) was defined as a ≤0.1% *BCR-ABL*/total *ABL* ratio. Treatment failure was defined as (1) inability to achieve any CyR at 6 months, (2) less than PCyR at 12 months or less than CCyR at 18 months, (3) disease relapse as indicated by a loss of CCyR or a confirmed one log increase of *BCR-ABL*/total *ABL* ratio, as well as (4) transformation to accelerated phase disease or blast crisis. In cases of treatment failure, tyrosine kinase domain mutation tests were indicated.

### Pharmacogenetic Analysis

Genomic DNA was extracted from 3 ml of peripheral blood samples using the Gentra® Puregene® extraction kit (Qiagen Inc, Minneapolis, MN, USA) and stored at −80°C until analyzed. Pharmacogenetic analyses involved screening and genotyping of polymorphic variants in the entire *SLC22A1* gene (UCSC RefSeq: NM_003057), spanning 40 kilobases, including 2 kilobases upstream and 1.5 kilobases downstream, on chromosome 6. Eleven candidate functional polymorphisms in *ABCB1*, *ABCG2*, *CYP3A5* and *PXR* genes which were described in our previous publications [30]–[33] were also investigated. *CYP3A4* polymorphisms were observed very rarely in Asian population in our previous studies [30], [34] and therefore not included in the present study. Data of healthy subjects from these publications is shown in Table S1. The PCR conditions employed are listed in Table S2. PCR amplicons were treated with Exonuclease I and Shrimp Alkaline Phosphatase before direct sequencing using the Applied Biosystems 3730 DNA Analyzer (Applied Biosystems Inc, CA, USA).

### Pharmacokinetic Analysis

Peripheral blood samples were taken from the patients 24 hours following their last IM dose. Steady state plasma trough concentrations of IM (C_0h_) were estimated via a validated high performance liquid chromatography (HPLC) method that was modified from a previously published technique [35]. Briefly, after mixing with 100 ng of 4-hydroxy-benzophenone as an internal standard [36], 50 µL plasma was deproteinized with 100 µL of methanol. After vortex-mixing, the mixture was centrifuged at 10,000× *g* at 4°C for 10 min, and 20 µl of the supernatant was subject to HPLC analysis. Chromatographic separation was accomplished using a C8 column (150 mm×4.6 mm I.D., 5 µm XTerra RP-8, Waters, USA) with a mobile phase consisting of 20 mM potassium dihydrogen phosphate:acetonitrile (72∶28, v/v). The lower limit of quantitation was 7.8 ng/mL. The calibration curve was linear over a concentration range of 7.8–8000 ng/mL. The within-day and between-day coefficients of variation were less than 10%. Imatinib clearance (CL) was calculated by the formula IM dosage/(dosing interval*C_0h_), whereby the IM dosage ranged from 300–600 mg and dosing interval was 24 hours. The final parameters used in the analyses on C_0h_ and CL were normalized by IM dosage.

### Statistical Analysis

The Hardy-Weinberg equilibrium between the genotypes was assessed using Fisher’s exact test. The Chi-square test was employed to assess the differences in genotype and allele distributions among the different groups in healthy subjects. Linkage disequilibrium (LD) analysis and LD block constructions were carried out using the software Haploview ver. 4.2 (Broad Institute, MA, USA) and quantified by |D′| and rho-square (r^2^) values. Tag-SNPs were identified by using Tagger program in Haploview software. Haplowalk method [37], [38] was applied to infer the haplotypes and generalized linear model (GLM) to estimate the individual haplotypic effect on IM pharmacokinetics. Haplotype specific GLM (haplo.glm) method is available under “haplostats” package in R-software to analyze the influence of haplotypes on a given trait (binary or quantitative) in unrelated subjects where haplotypes are often ambiguous because of unknown linkage phase of the measured sites along a chromosome. To assign the haplotypes copy number for each patient, haplotype phasing was conducted using PLINK 1.02 v. Association of genotype and haplotype copy numbers with IM pharmacokinetics was evaluated by Mann-Whitney U or Kruskal-Wallis tests, as indicated. Based on our results, we assessed the combinatorial effect of *SLC22A1* and *ABCB1* haplotype profiles on the prediction of IM pharmacokinetics. Imatinib trough level association with clinico-demographic characteristics and response parameters was evaluated with Mann-Whitney U test for categorical data and Kendall Tau correlation test for continuous data. All tests were performed using SPSS statistics software ver. 18 (IBM, Chicago, IL, USA). All statistical evaluations were made assuming a two-sided test with significance level of 0.05 unless otherwise stated.

## Results

### Patient Demographics

The median age at diagnosis and BMI of the patients were 42 years (range: 16–69) and 24.4 kgm^−2^ (range: 17.9–49.5), respectively. All patients received IM for at least 18 months, starting at a daily dose of 400 mg. At the point of recruitment, 4 patients were on an escalated daily dose of 600 mg as they could not achieve major molecular response, and 2 were on an attenuated dose of 300 mg due to toxicity issues. Median follow-up time from the start of IM was 101.6 months (range 25.6–132.7). All patients achieved complete hematological response within 3 months of IM treatment. At 6 months, one patient had no CyR and was considered to have failed treatment, whereas the remainder had achieved minor CyR (n = 2), PCyR (n = 10) or CCyR (n = 24), or remained unknown (n = 1). At 12 months, no patients were identified to have achieved less than PCyR except for the one who had failed treatment earlier. At 18 months, all patients achieved CCyR except for 3 who were considered to have failed treatment. Of 28 patients with available data for molecular response at 18 months, 14 patients did not achieve MMR and were considered to have suboptimal response to IM treatment. In the course of follow-up post-IM therapy, one patient eventually relapsed at 56.5 months whilst another patient succumbed to blast crisis at 90.7 months. Upon further analysis, non-synonymous mutations were detected in the *BCR-ABL* kinase domain in these two patients - a double mutation at loci G250E and E255V in the former patient and a single mutation at Y253H in the latter patient. No mutations were detected in the remaining three patients. Overall, a total of five patients (13.2%) were considered to have failed treatment.

The median time from diagnosis to IM plasma level testing was 71.5 months, and the median time from the commencement of IM therapy to plasma level testing was 58.4 months. Trough IM plasma levels were highly variable, ranging from 625 to 5271 ng/mL, with mean (±SD) and median levels of 1959 (±1073) and 1719 ng/mL, respectively. For patients on the standard IM dose of 400 mg (n = 32), the mean (±SD) and median levels were 1899 (±1054) and 1395 ng/mL, respectively. Patients on the escalated dose of 600 mg (n = 4) had higher mean (±SD) and median levels of 2785 (±1093) and 2384 ng/mL, respectively, although this was not statistically significant. Notably, we observed that out of 34 patients on ≤400 mg dose of IM, 31 (94%) had an IM trough concentration of >1000 ng/mL (range: 679–5272 ng/mL) (Table 2). None of the patients with an IM trough of <1000 ng/mL failed treatment. The IM trough levels of the 5 patients who failed treatment were 2873, 1372, 1162, 1177 and 2633 ng/mL, respectively. IM trough levels were not significantly associated with patient clinico-demographic characteristics such as body weight, body mass index, body surface area, gender and age, nor with any of the response parameters (Table 2).

**Table 2:** Clinical correlates with imatinib trough in patients on 400 mg standard dose.

Characteristics	n	IM Trough (ng/mL)			*SLC22A1* haplotype profile		
		Mean±SD	Median (range)	*p*-value^a^	S_high_	S_low_	*p*-value^b^
**Total**	32	1899±1054	1395 (678–5272)	–	–	–	
**Gender**					–	–	–
Male	19	1944±1137	1417 (678–5272)	0.863	12	7	1.000
Female	13	1833±959	1365 (714–4219)		8	5	
**Treatment response**							
CCyR 6 months							
No	11	2017±800	1881 (1108–3278)	0.505	8	3	0.466
Yes	19	1896±1226	1372 (678–5272)		11	8	
CCyR 12 months							
No	4	2289±964	2359 (1161–3278)	0.312	4	0	0.268
Yes	25	1857±1111	1372 (678–5272)		15	10	
MMR 18 months							
No	11	1828±936	1506 (625–4219)	0.260	7	4	0.080
Yes	10	1974±1375	1364 (678–4272)		2	8	
Treatment failure							
No	28	1935±1090	1506 (678–5272)	0.894	16	12	0.265
Yes	3	1802±933	1372 (1161–2873)		3	0	
**Continuous variables**		**Mean±SD**	**Median (range)**	***p*** **–value^c^**			
Age (years)		49±12	48 (23–79)	0.486	–	–	–
Body weight (kg)		67.5±15.6	65.5 (47–135.5)	0.838	–	–	–
Height (cm)		164.2±7.8	165 (145–180)	0.183	–	–	–
BMI (kg/m^2^)		25.1±5.7	24.4 (17.9–49.5)	0.285	–	–	–
BSA (m^2^)		1.8±0.2	1.7 (1.4–2.5)	0.769	–	–	–

Associations of clinico-demographic characteristics and response parameters with imatinib trough and *SLC22A1* haplotypes were evaluated statistically. Abbreviations: CCyR, complete cytogenetic response; MMR, major molecular response; BMI, body mass index; BSA, body surface area.

a, b, c *p*-values were calculated using ^a^Mann-Whitney, ^b^Fisher exact and ^c^Kendall Tau correlation tests, respectively.

### *SLC22A1* Polymorphisms in Healthy Asians

Complete screening of the *SLC22A1* gene was first performed on healthy subjects of Chinese, Malay and Indian ethnicity. Table S1 summarizes the genotypic and allelic frequencies in healthy Asian subjects. A total of 89 SNPs were identified whereby 4 were located in the 5′-upstream region, 7 in the exonic region and 78 in the intronic region. Fig. S1 illustrates the *SLC22A1* gene structure and locations of these SNPs. Fisher’s exact test showed that *SLC22A1* genotype frequencies at all loci conformed to Hardy-Weinberg equilibrium except for a single intronic SNP IVS1+556G>A (rs62440864) in the Malay population (*p*<0.001). Of these 89 SNPs, 20 were found to be monomorphic in Chinese, 9 in Malays and 11 in Indians. Six SNPS [IVS2-1010T>G (rs614890), 1222A>G (rs628031), IVS8+4215T>C (rs654993), IVS8+5402T>C, IVS8-2964C>A (rs622342), and IVS8-1295C>A (rs650284)] were found to be highly polymorphic in all ethnic groups (variant allele frequency range, 52% to 85%). Statistically significant interethnic differences in the genotypic distributions among three ethnic groups were detected for 34% (n = 30) of the polymorphisms (Table S1, *p*<0.016).

### LD and Tag-SNPs Analysis in Healthy Asians

**Figure 1:**
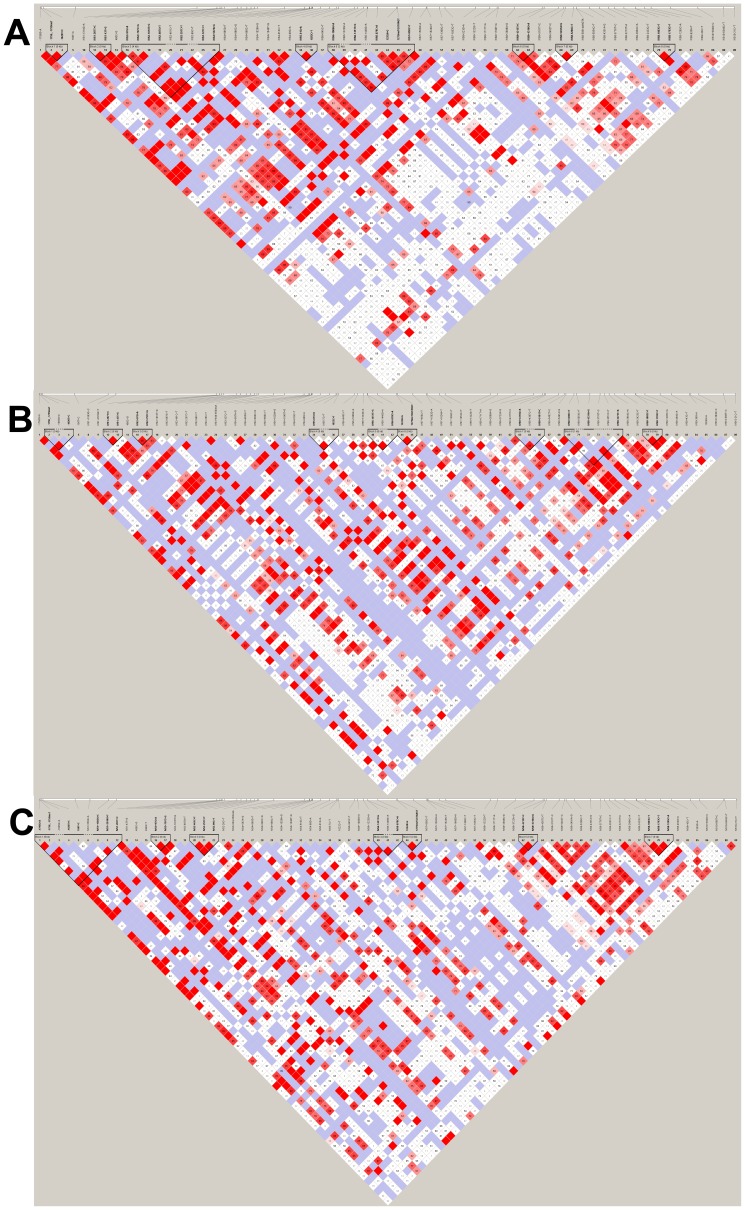
Linkage disequilibrium plots of *SLC22A1* polymorphisms in healthy Asians. Pairwise LD matrices represent moderate to strong linkage between *SLC22A1* polymorphisms among (A) Chinese, (B) Malay and (C) Indian ethnic groups.

Pairwise LD analyses were performed between the 89 SNPs identified earlier for each of the ethnic groups. Pairwise LD matrices demonstrated moderate to strong linkage between several SNPs throughout the *SLC22A1* gene region. Tight linkage was observed amongst the following SNPs across all ethnicities: IVS5-61G>A (rs2282142) with 1022C>T (rs2282143), IVS2-687G>T (rs3798175) and IVS4+1028A>G (rs3798170) (|D′| >0.90, r^2^>0.85); IVS2+97G>A (rs4646273) and IVS2+797C>G (rs4646274) with IVS4+1040T>G (rs3798169) and IVS7-1368C>T (rs1867350) (|D′| >0.94, r^2^>0.82); IVS2-461C>T (rs3798174) with IVS2-99C>T (rs3737088), IVS4+886C>G (rs3798172) and IVS7-1053C>T (rs7766568) (|D′| >0.90, r^2^>0.80); IVS2-257C>T (rs4646275) with IVS4+886C>G (rs3798172), IVS4-98G>A (rs4646276) and IVS7-1053C>T (rs7766568) (|D′| >0.90, r^2^>0.80); IVS2-1010T>G (rs614890) with IVS4+597A>G (rs594709) (|D′| = 1, r^2^>0.91) located in the region spanning introns 2–7; 5′-upstream region SNPs were in moderate linkage with intronic SNPs, -1756_-1755insT and -1620T>C (rs9457840) with IVS2-687G>T (rs3798175), IVS4+1028A>G (rs3798170) and IVS5-61G>A (rs2282142) (|D′| >0.90, r^2^>0.80); −1795G>A (rs6935207) with IVS1-207T>C (rs9457841) and IVS1-43T>G (rs4646272) (|D′| >0.80, r^2^>0.52); introns 8 and 9 polymorphisms, IVS8+1322G>T (rs644992) with IVS8+2698A>G (rs637841) (|D′| >0.96, r^2^>0.90); IVS8+5101T>C (rs7750592) with IVS8-4331A>G (rs9347386) (|D′| >0.91, r^2^>0.80) and IVS8-1803C>T (rs4709401) with IVS8-1793C>T (rs4709402) and IVS9+43C>T (rs2297374) (|D′| >0.92, r^2^>0.82). A detailed LD pattern is shown in Fig. 1 A–C where LD matrices are displayed by Chinese, Malay and Indian ethnic groups, respectively. Tag-SNPs were selected to represent the entire *SLC22A1* region for each ethnic group by using pairwise tagging method in the Tagger program (Haploview 4.2, Broad institute USA) as described by de Bakker *et al*. [39]. A total of 24 tag-SNPs were selected to represent the 89 SNPs in all ethnicities, which included 2 5′-upstream, 2 exonic and 20 intronic SNPs (Table S1, see footnotes).

### Pharmacogenetic Screening in CML Patients

Pharmacogenetic screening of the selected 24 *SLC22A1* tag-SNPs, as well as candidate SNPs from *ABCB1* [1236C>T (rs1128503), 2677G>T/A (rs2032582), 3435C>T (rs1045642)], *ABCG2* 421C>A (rs2231142), CYP3A5*3 (rs776746) and *PXR* [IVS2+55A>G (rs1464603), IVS2+78A>G (rs1464602), IVS6-17C>T (rs2276707), 1792A>G (rs3732359), 1944T>C (rs3732360), 2654T>C (rs3814058)] was performed in 38 CML patients. The genotypic and allelic frequencies of *SLC22A1* tag-SNPs in the patient cohort were found to be similar to that observed in the healthy Chinese subjects as majority of patients were of Chinese descent. Candidate SNPs selected from *ABCB1, ABCG2, CYP3A5* and *PXR* genes were present with high variant allele frequency ranging from 20% to 70% in the patients with CML which was also comparable with healthy subjects from the 3 ethnic groups (Table S1). None of the individual genotypes demonstrated a significant association with IM pharmacokinetic parameters (data not shown). Haplotype analysis have been recognized to be more robust than single marker analysis [40], [41] and these finding supported the hypothesis to look into multi-marker interactions to reveal the genotypic-phenotypic association. The 24 *SLC22A1* tag-SNPs were considered for haplotypes and IM pharmacokinetic association analysis in the CML patients.

### Haplotypic Effect of *SLC22A1* Tag-SNPs on IM Pharmacokinetics

The haplotypic effect of *SLC22A1* tag-SNPs on IM pharmacokinetics was evaluated using the *haplowalk* method. This method was adapted to identify a sub-haplotypic region that could best explain the variability in IM pharmacokinetic parameters. Global haplotype score statistics was applied to estimate the overall association of haplotypes and phenotypic traits [37], [38]. Our analysis revealed a sub-haplotypic region encompassing one exonic SNP [1222A>G (rs628031)] surrounded by two intronic SNPs [IVS6-878C>A (rs3798168) and IVS7+850C>T] that is significantly associated with IM clearance (*p* = 0.013, adjusted for gender, weight, age and dosing interval, Fig. S2 **)**. The haplowalk score statistic displays a global overview of the association of haplotypes in a particular region with phenotypic traits, and cannot be interpreted as a specific individual haplotypic effect. Therefore, we further employed the haplotype-specific GLM in order to estimate the individual haplotypic effects.

**Figure 2:**
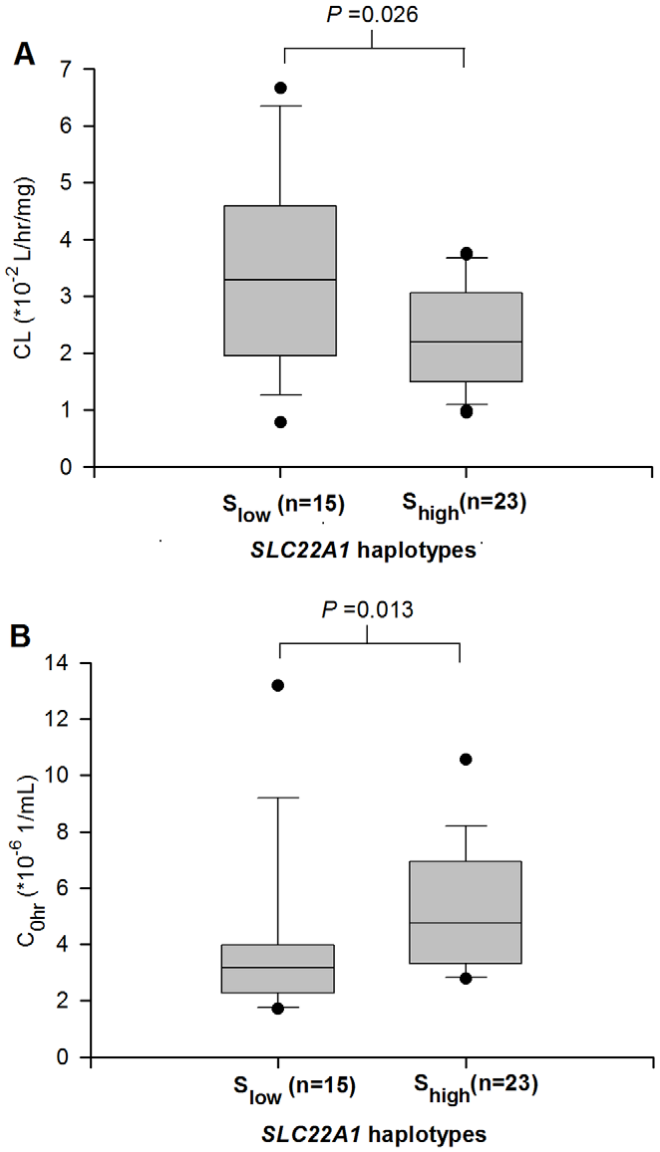
*SLC22A1* haplotypes association with imatinib pharmacokinetics in Asian patients with CML (n = 38). The patients were divided in two groups according to copy numbers of *SLC22A1* haplotypes AGT and CGC, S_low_ (0 or 1 copy) and S_high_ (2 copies). *SLC22A1* haplotypes were significantly associated with imatinib (A) clearance, CL and (B) trough concentration, C_0h._ Patients harboring S_high_ haplotypes had 33.4% lower clearance and 50% higher trough concentration than patients with S_low_ haplotypes_._

Haplotype-specific GLM estimates the regression coefficient associated with IM pharmacokinetics (Table 3). The high frequency haplotypes (IVS6-878C>A; 1222A>G; IVS7+850C>T) AGT and CGC (frequency: 41% and 36%, respectively) were significantly associated with a 22% decrease in IM clearance compared to the reference haplotype CAC (frequency: 22%) [CL (*10^−2^ L/hr/mg); CAC vs AGT: 4.03 vs 3.16, *p* = 0.017; CAC vs CGC: 4.03 vs 3.15, *p* = 0.017]. The *SLC22A1* haplotype AGC was found to be present in only 1% of our CML patients, however the IM clearance was observed to be 1.7-fold higher than reference haplotype [CL (*10^−2^ L/hr/mg); CAC vs AGC: 4.03 vs 6.71, *p* = 0.04]. Similarly, haplotypes AGT and CGC were modestly associated with a 38% and 30% increase in C_0h_, respectively, as compared to reference haplotype CAC, although this was not statistically significant [C_0h_ (*10^−6^ 1/ml); CAC vs AGT: 3.13 vs 4.32; CAC vs CGC: 3.13 vs 4.07; *p*>0.05]. As both of these low clearance-associated haplotypes had similar impact on IM disposition, we examined the combinatorial effect of copy numbers of these two haplotypes (AGT and CGC) on IM pharmacokinetics. Two copies of haplotypes AGT or CGC were observed in 23 patients, 1 copy in 12 patients whereas 3 patients were not carrying any copies of AGT or CGC haplotypes. The patients were divided in two groups according to the number of copies of haplotypes AGT or CGC. The first group (n = 15) comprised of patients carrying 0 or 1 copy (S_low_) and the second group (n = 23) comprised of patients carrying 2 copies of AGT or CGC haplotypes (S_high_). The copy numbers of AGT and CGC haplotypes were significantly associated with IM clearance and C_0h_ (Fig. 2). Patients harboring S_high_ had 33.4% lower clearance and 50% higher C_0h_ than patients with S_low_ [median clearance (*10^−2^ L/hr/mg); S_low_ vs S_high_: 3.29 vs 2.19, *p* = 0.026; median C_0h_ (*10^−6^ 1/ml); S_low_ vs S_high_: 3.17 vs 4.76, *p* = 0.013].

**Table 3:** Influence of *SLC22A1* haplotypes on imatinib clearance, CL and trough concentration, C_0h_ using haplotype specific generalized linear model.

Haplotypes			Haplotype frequency (%)		Mean ± SE		Fold change		*p*-value	
IVS6 -878C>A	1222A>G	IVS7+850C>T	CMLPatients	Healthy Chinese	CL(*10^−2^ L/hr/mg )	C_0h_(*10^−6^ 1/ml)	CL	C_0h_	CL	C_0h_
C	A	C	22.3	25.8	4.03±0.53	3.13±1.06	1.00^a^	1.00^a^	0	0.005
A	G	C	1.30	0.90	6.71±1.28	0.60±2.56	1.66	0.192	0.044	0.328
A	G	T	40.7	38.7	3.16±0.35	4.32±0.70	0.784	1.38	0.017	0.097
C	G	C	35.5	30.2	3.15±0.35	4.07±0.71	0.781	1.30	0.017	0.192

a Reference: Imatinib pharmacokinetics parameters values corresponding to haplotype CAC were used as reference to compare with other three haplotypes.

### Effect of a Combined *SLC22A1-*ABCB1 Haplotype Profile on IM Pharmacokinetics

**Figure 3:**
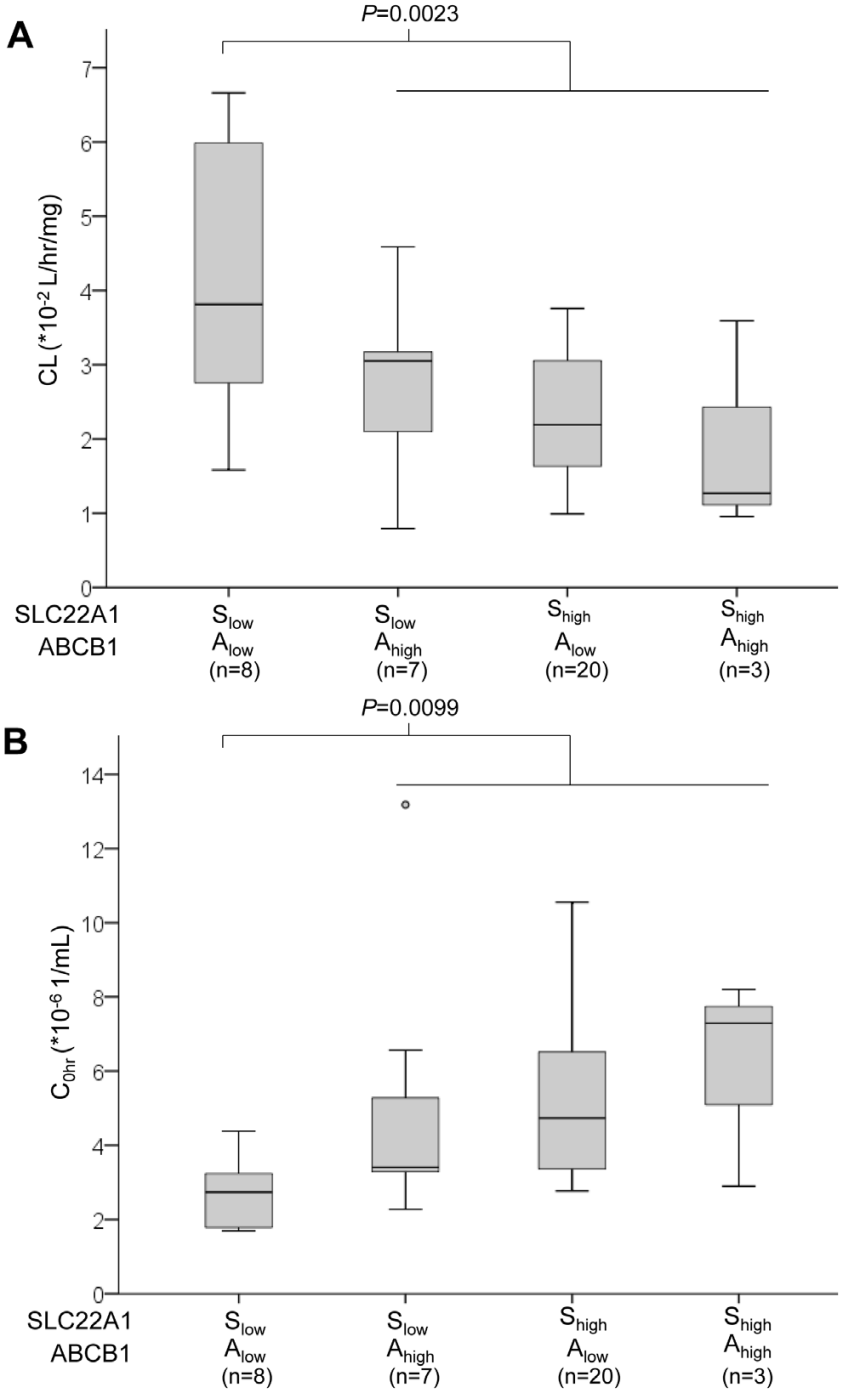
*SLC22A1* and *ABCB1* haplotypes association with imatinib pharmacokinetics in Asian patients with CML (n = 38). *SLC22A1 and ABCB1* haplotypes were stratified according to specific combination haplotype profiles and associations were checked with imatinib (A) clearance, CL and (B) trough concentration, C_0h_. A trend towards low clearance and high C_0h_ was observed from S_low_-A_low_, S_low_-A_high_, S_high_-A_low_ to S_high_-A_high_. The simultaneous possession of S_low_ with A_low_ was associated with a 73.2% higher clearance and 41.2% lower trough concentration than patients carrying other haplotype combinations.

We explored if specific *ABCB1* haplotypes could affect IM clearance and concentrations. Interestingly, although we did not find any direct correlations amongst *ABCB1* haplotypes with clearance and C_0h_, we observed that patients harboring S_high_ tended to carry either 0 or 1 copy of *ABCB1* haplotypes (1236C>T; 267G>T/A; 3435C>T) TGT or TTT (A_low_), rather than 2 copies (A_high_) (*p* = 0.030). When stratified according to specific *SLC22A1-ABCB1* combination haplotype profiles, a trend towards low clearance and high C_0h_ was observed from S_low_-A_low_, S_low_-A_high_, S_high_-A_low_ to S_high_-A_high_. Notably, the simultaneous possession of S_low_ with A_low_ was associated with a 73.2% higher clearance and 41.2% lower C_0h_ than patients carrying other haplotype combinations [median CL (*10^−2^ L/hr/mg); S_low_-A_low_ vs others: 3.81 vs 2.20, *p* = 0.0023; median C_0h_ (*10^−6^ 1/ml); S_low_-A_low_ vs others: 2.74 vs 4.66, *p* = 0.0099] (Fig. 3). The combined *SLC22A1-ABCB1* haplotype profile was not significantly associated with treatment response parameters.

## Discussion

Our results demonstrated wide inter-individual variability of trough IM plasma levels in Asian patients with CML, confirming previous reports on Caucasian [3], [6], [10], [16], Korean [13], Japanese [12], Chinese [11], [17] and other ethnic cohorts [14], [15], [18]. In addition, we not only observed that the mean IM trough concentration in our cohort of Asian patients with CML was significantly higher than that previously reported in studies on Caucasians [3], [6], [10], [14], [16], [18], 31 of 34 patients (94%) who received standard IM doses of 400 mg in fact had an IM trough concentration above the recommended threshold of >1000 ng/mL. This result is consistent with studies on the Chinese [11], [17], [42], Korean [13] and Jordanian [15] populations. These differences in IM levels may be attributed to several factors including poor medication compliance [6], intake of other drugs [7], gender as well as physical variables [3], [17]. In our study however, there was no evidence that any of the above factors could have accounted for the large inter-patient and inter-ethnic variability observed. Instead, the data indicated that the underlying reason may invoke intrinsic differences in cellular influx (*e.g.* hOCT1) and efflux (*e.g.* ABCB1) transporters [9]. hOCT1 is known to mediate the active transport of IM into primary CML cells [20], and is likely to be a major determinant of hepatocyte uptake for systemic clearance [43]. Low hOCT1 or high ABCB1 activity/expression in leukemic cells may result in reduced influx or increased efflux of drugs respectively, thereby reducing intracellular levels whilst increasing plasma levels. Simultaneously, the same effect on hepatocytes may impair drug uptake into the liver, leading to elevated plasma levels. Whilst it may be plausible that since some patients with CML achieve supra-therapeutic IM levels on standard 400 mg doses and may be considered for de-escalated regimens without aggravating their outcome [17], such observations may also reflect compromised cellular transport mechanisms resulting in high plasma levels and detrimentally-low intracellular levels.

In this exploratory study, we screened the entire *SLC22A1* gene for polymorphic variation in three healthy Asian ethnic populations and further examined tag-SNPs in Asian CML patients. Our approach revealed a sub-haplotypic region encompassing 1 exonic SNP [1222A>G (rs628031)] surrounded by 2 intronic SNPs [IVS6-878C>A (rs3798168) and IVS7+850C>T] that is significantly associated with IM pharmacokinetics. The *SLC22A1* gene was found to be highly polymorphic and displayed significant interethnic variations amongst healthy individuals of Chinese, Malay, and Indian ethnicity (Table S1, see footnotes). For example, the allelic frequency of the 5′-upstream polymorphism [−1795G>A (rs6935207)], was significantly higher amongst Chinese (0.57) as compared to Malays (0.39), Indians (0.30) and Caucasians (0.21) [26]. Interestingly, the majority of previously studied *SLC22A1* coding SNPs in Caucasians [24], [25], [27] were absent in the Asian ethnic groups examined, and only 1222A>G (rs628031) was found in common amongst Caucasian, Japanese [44] and the current Asian cohort. Similarly, polymorphisms 181C>T (rs12208357) and 1260_1262delGAT (rs72552763) which have been linked to reduced transport activity of hOCT1 substrates in European-American groups [45], were not observed in Asians.

Amongst our cohort of Asian patients with CML, we were able to identify two groups based on unique haplotype profiles - one composed of those harboring 0 or 1 copy (S_low_) and the other carrying 2 copies of AGT or CGC haplotypes (S_high_). Patients harboring S_high_ had 50% higher IM trough level and 33.4% lower clearance than patients with S_low_. The haplotype CGC was represented by the presence of the wild type allele of intronic polymorphisms IVS6-878C>A (rs3798168) and IVS7+850C>T, as well as the variant allele of the non-synonymous SNP 1222A>G (rs628031). On the other hand, the haplotype AGT was represented by the presence of the variant allele of all three polymorphisms. To the best of our knowledge, the intronic polymorphisms IVS6-878C>A (rs3798168) and IVS7+850C>T were not studied with phenotypic data previously. Although IM is a substrate of hOCT1 [20], it has been shown in previous single marker studies that 1222A>G (rs628031) does not alter the function of the hOCT1 protein [46] and does not affect response to IM therapy in CML patients [12], [24], [26], [27]. Our positive results from haplotype analysis is unsurprising, given that such an approach is known to be more robust than single marker analysis for identifying genomic regions enriched for phenotype-relevant casual variants [40], [41]. In the present study, a moderate to strong linkage pattern was observed amidst *SLC22A1* variants in different ethnic groups. Particularly, strong linkage (|D′| >0.90) was detected amongst these three key variants in the Chinese population, which formed 84.2% of our patient cohort. Hence, the linkage effect of the region may have been the confounding factor in previous studies. The sub-haplotypic region is composed of two intronic and one coding SNP which suggests that the influence of haplotypes on IM pharmacokinetics could be mediated through interactions involving post-transcriptional modification. Taken together, out results highlight the importance of haplotype over single marker analysis to explain the variability in IM disposition and warrant further investigations in other ethnic groups.

Interestingly, we found that *ABCB1* haplotypes may exert an indirect modulatory effect upon IM pharmacokinetics, depending on the specific *SLC22A1* haplotype, with a trend towards high IM trough levels being observed from S_low_-A_low_, S_low_-A_high_, S_high_-A_low_ to S_high_-A_high_ (Fig. 3). Previous studies focusing only on genotype rather than haplotype profiles obtained heterogeneous results, with Gurney *et al.* reporting a lower IM clearance in individuals with the *ABCB1* 1236CC, 2677GG or 3435CC genotypes [47], Yamakawa *et al.* demonstrating higher clearance in Japanese patients with the 3435CC genotype [28], and others reporting a lack of any association [12], [48]. In agreement with our finding though, Dulucq *et al.* showed that both *ABCB1* haplotype TTT as well as genotype 1236TT were correlated with high IM trough levels [49]. These inconsistencies may perhaps be explained by a functional dependency of *ABCB1* on *SLC22A1*. In support of this thinking, it was noted in our study that patients with the *SLC22A1* haplotype S_high_ tended to possess the *ABCB1* haplotype A_low_, whilst a previous microarray analysis on a panel of leukemic cell lines revealed that *SLC22A1* was inversely related with gene expression of *ABCB1* [50]. This implies that specific *ABCB1* and *SLC22A1* haplotype profiles may affect IM levels by affecting their gene expression.

In conclusion, the present exploratory study comprehensively screened for *SLC22A1* genetic variations and investigated its linkage and haplotype pattern in three distinct Asian populations, followed by its association with IM pharmacokinetics in CML patients. It is acknowledged that there are limitations in the current study and the results should be interpreted with care. Nonetheless, our report is the first to suggest that genetic polymorphisms and specific haplotypes in *SLC22A1* and *ABCB1* may contribute towards inter-individual and inter-ethnic variations in IM disposition in CML patients. The validation, as well as generalizability of results awaits future investigations in larger cohorts, including in other ethnic groups. More importantly, the functional and biological characterization of the haplotype variants ought to be further investigated.

## Supporting Information

Figure S1 ***SLC22A1*** **gene structure and location of single nucleotide polymorphisms.** Figure represents the 89 identified polymorphisms in the *SLC22A1* gene. Four polymorphisms were located in the 5′-upstream region, 7 in the exonic region and 78 in the intronic region.
(TIF)

Figure S2 **Haplowalk Analysis.** Haplowalk analysis showing the sub-haplotypic region associated with imatinib clearance. *P*-values corresponding to polymorphisms are plotted on –log^10^ base. The sub-haplotypic region, significantly associated with imatinib clearance, is shown in blue dotted box. This region encompasses one exonic SNP (rs628031) surrounded by two intronic SNPs (rs3798168 and IVS7+850C>T).
(TIF)

Table S1 Genotype and allele frequency of *SLC22A1, ABCB1, ABCG2, CYP3A5* and *PXR* single nucleotide polymorphisms among Asian healthy subjects (Chinese, Malay and Indian, n = 70 each) and CML patients (n = 38). *SLC22A1* 24 Tag SNPs and statistically significant interethnic differences in the genotypic distributions among healthy subjects of three ethnic groups are indicated by table footnotes. Table also represents *ABCB1, ABCG2, CYP3A5* and *PXR* gene polymorphism data from our previous publications [30]–[33].
(DOC)

Table S2 Primer Sequences and PCR Conditions for Amplifications of *SLC22A1* regions (UCSC RefSeq: NM_003057).
(DOC)
