# Supplementary figures and images for: SLC22A1-ABCB1 Haplotype Profiles Predict Imatinib Pharmacokinetics in Asian Patients with Chronic Myeloid Leukemia
Source: PLoS One. 2012 Dec 18;7(12):e51771. doi: 10.1371/journal.pone.0051771 (PMC3525665; doi:10.1371/journal.pone.0051771)

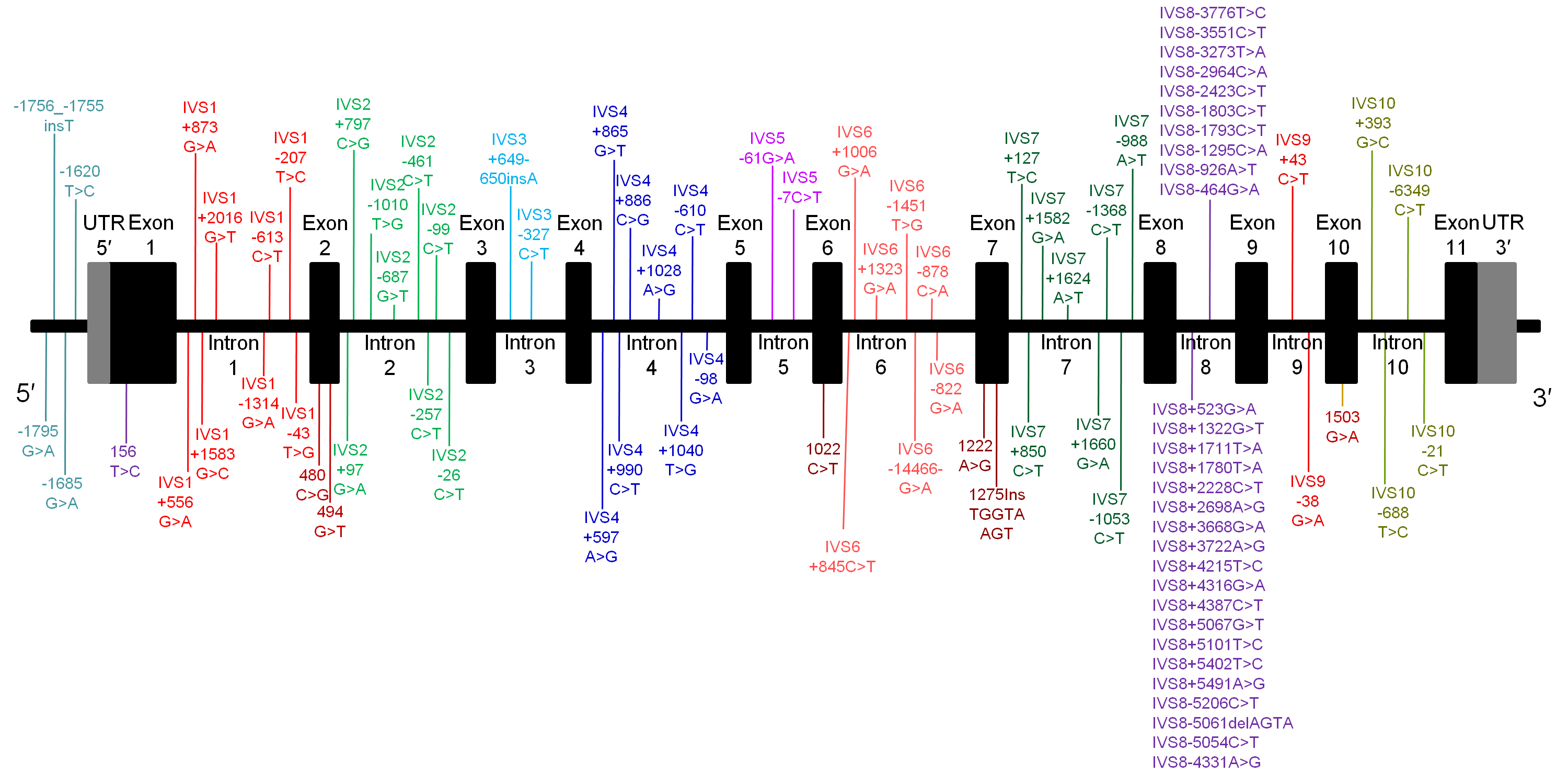

Supplement: Figure S1 — SLC22A1 gene structure and location of single nucleotide polymorphisms. Figure represents the 89 identified polymorphisms in the SLC22A1 gene. Four polymorphisms were located in the 5′-upstream region, 7 in the exonic region and 78 in the intronic region. (TIF) [file pone.0051771.s001.tif]

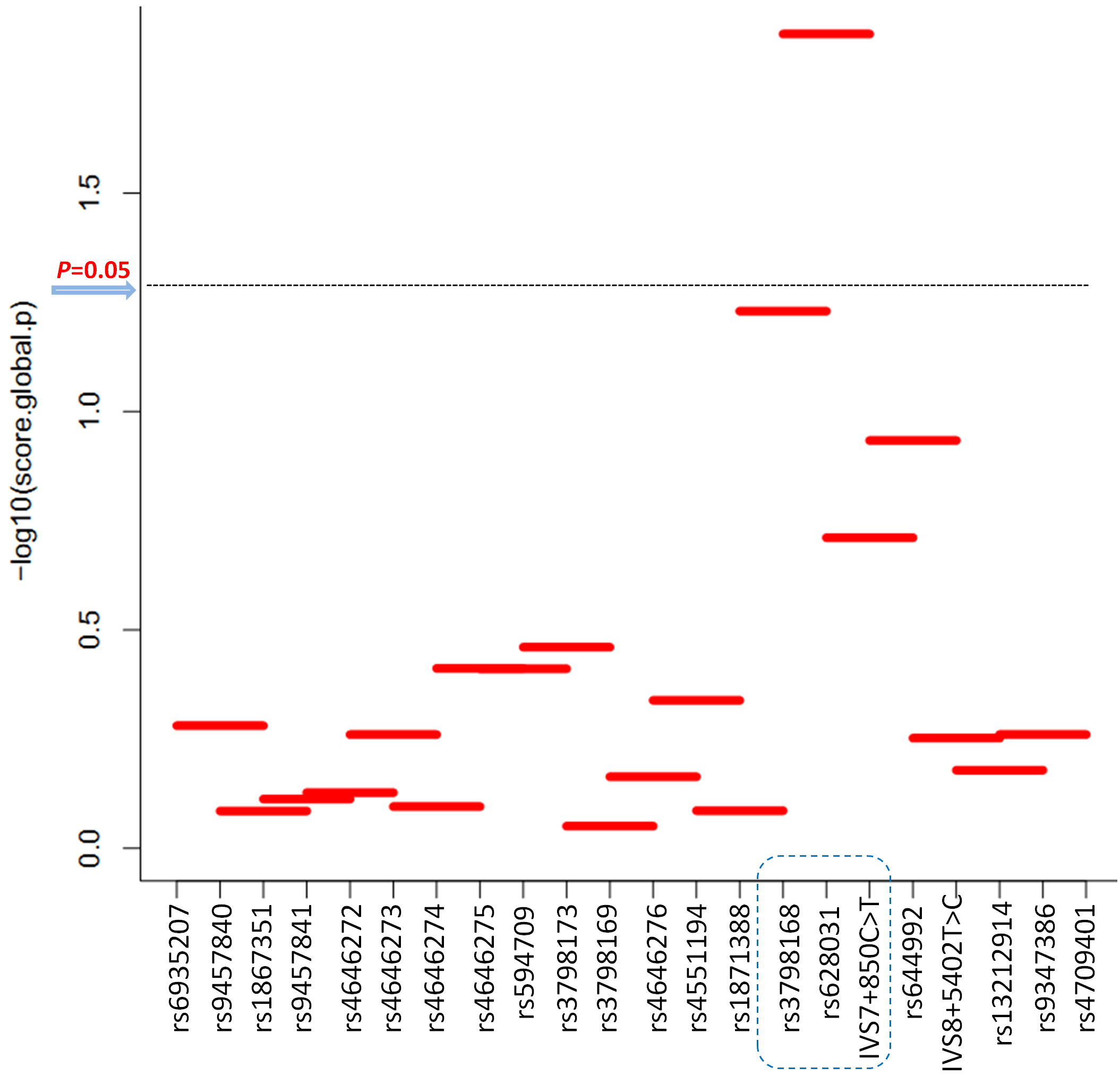

Supplement: Figure S2 — Haplowalk Analysis. Haplowalk analysis showing the sub-haplotypic region associated with imatinib clearance. P-values corresponding to polymorphisms are plotted on –log10 base. The sub-haplotypic region, significantly associated with imatinib clearance, is shown in blue dotted box. This region encompasses one exonic SNP (rs628031) surrounded by two intronic SNPs (rs3798168 and IVS7+850C>T). (TIF) [file pone.0051771.s002.tif]
