# Supplementary material for: SLC22A1-ABCB1 Haplotype Profiles Predict Imatinib Pharmacokinetics in Asian Patients with Chronic Myeloid Leukemia
Source: PLoS One. 2012 Dec 18;7(12):e51771. doi: 10.1371/journal.pone.0051771 (PMC3525665; doi:10.1371/journal.pone.0051771)
Supplement: Table S1 — Genotype and allele frequency of SLC22A1, ABCB1, ABCG2, CYP3A5 and PXR single nucleotide polymorphisms among Asian healthy subjects (Chinese, Malay and Indian, n = 70 each) and CML patients (n = 38). SLC22A1 24 Tag SNPs and statistically significant interethnic differences in the genotypic distributions among healthy subjects of three ethnic groups are indicated by table footnotes. Table also represents ABCB1, ABCG2, CYP3A5 and PXR gene polymorphism data from our previous publications [30]–[33]. (DOC) [file pone.0051771.s003.doc]

**Table S1.** Genotype and allele frequency of *SLC22A1, ABCB1, ABCG2, CYP3A5* and *PXR* single nucleotide polymorphisms among Asian healthy subjects (Chinese, Malay and Indian, n=70 each) and CML patients (n=38).

| **SN** | **SNPs** | **rs ID** | **Genotype** | **Genotype frequency, n (%)** | | | | **Allele** | **Allele Frequency (95%confidence interval)** | | | |
| --- | --- | --- | --- | --- | --- | --- | --- | --- | --- | --- | --- | --- |
|  |  |  |  | **Chinese** | **Malays** | **Indians** | **CML Patients** |  | **Chinese** | **Malays** | **Indians** | **CML Patients** |
|  | ***SLC22A1*** |  |  |  |  |  |  |  |  |  |  |  |
| **1** | **-1795G>A^a,b^** | rs6935207 | GG | 14 (20) | 25 (35.71) | 35 (50) | 8 (21.1) | **G** | 0.43 (0.35-0.51) | 0.61 (0.53-0.69) | 0.7 (0.62-0.78) | 0.46 (0.35-0.57) |
|  |  |  | GA | 32 (45.71) | 36 (51.43) | 28 (40) | 19 (50) | **A** | 0.57 (0.49-0.65) | 0.39 (0.31-0.47) | 0.3 (0.22-0.38) | 0.54 (0.43-0.65) |
|  |  |  | AA | 24 (34.29) | 9 (12.86) | 7 (10) | 11 (28.9) |  |  |  |  |  |
| **2** | **-1756_-1755insT** |  | wt | 53 (75.71) | 58 (82.86) | 57 (81.43) |  | **wt** | 0.88 (0.82-0.93) | 0.91 (0.87-0.96) | 0.89 (0.84-0.94) |  |
|  |  |  | wt/ins | 17 (24.29) | 12 (17.14) | 11 (15.71) |  | **ins** | 0.12 (0.07-0.18) | 0.09 (0.04-0.13) | 0.11 (0.06-0.16) |  |
|  |  |  | ins | 0 | 0 | 2 (2.86) |  |  |  |  |  |  |
| **3** | **-1685G>A** | rs112785810 | GG | 70 (100) | 68 (97.14) | 67 (95.71) |  | **G** | 1 | 0.96 (0.93-0.99) | 0.98 (0.95-1) |  |
|  |  |  | GA | 0 | 2 (2.86) | 3 (4.29) |  | **A** | 0 | 0.04 (0.01-0.07) | 0.02 (0-0.05) |  |
|  |  |  | AA | 0 | 0 | 0 |  |  |  |  |  |  |
| **4** | **-1620T>C^a^** | rs9457840 | TT | 53 (75.71) | 58 (82.86) | 57 (81.43) | 30 (78.9) | **T** | 0.88 (0.82-0.93) | 0.91 (0.87-0.96) | 0.89 (0.84-0.94) | 0.88 (0.81-0.95) |
|  |  |  | TC | 17 (24.29) | 12 (17.14) | 11 (15.71) | 7 (18.4) | **C** | 0.12 (0.07-0.18) | 0.09 (0.04-0.13) | 0.11 (0.06-0.16) | 0.12 (0.05-0.19) |
|  |  |  | CC | 0 | 0 | 2 (2.86) | 1 (2.6) |  |  |  |  |  |
| **5** | **156T>C^a,b^** | rs1867351 | TT | 19 (27.14) | 34 (49.28) | 45 (64.29) | 13 (34.2) | **T** | 0.55 (0.47-0.63) | 0.71 (0.63-0.79) | 0.81 (0.74-0.87) | 0.58 (0.47-0.69) |
|  |  |  | TC | 39 (55.71) | 30 (43.48) | 23 (32.86) | 18 (47.4) | **C** | 0.45 (0.37-0.53) | 0.29 (0.21-0.37) | 0.19 (0.13-0.26) | 0.42 (0.31-0.53) |
|  |  |  | CC | 12 (17.14) | 5 (7.25) | 2 (2.86) | 7 (18.4) |  |  |  |  |  |
| **6** | **IVS1+556G>A^b^** | rs62440864 | GG | 69 (100) | 63 (96.92) | 62 (93.94) |  | **G** | 1 | 0.97 (0.94-1) | 0.97 (0.94-1) |  |
|  |  |  | GA | 0 | 0 | 4 (6.06) |  | **A** | 0 | 0.03 (0-0.06) | 0.03 (0-0.06) |  |
|  |  |  | AA | 0 | 2 (3.08) | 0 |  |  |  |  |  |  |
| **7** | **IVS1+873G>A** |  | GG | 69 (98.57) | 65 (100) | 66 (100) |  | **G** | 0.99 (0.98-1.01) | 1 | 1 |  |
|  |  |  | GA | 1 (1.43) | 0 | 0 |  | **A** | 0.01 (-0.01-0.02) | 0 | 0 |  |
|  |  |  | AA | 0 | 0 | 0 |  |  |  |  |  |  |
| **8** | **IVS1+1583G>C^b^** | rs73025537 | GG | 70 (100) | 53 (89.83) | 51 (75) |  | **G** | 1 | 0.95 (0.91-0.99) | 0.86 (0.8-0.92) |  |
|  |  |  | GC | 0 | 6 (10.17) | 15 (22.06) |  | **C** | 0 | 0.05 (0.01-0.09) | 0.14 (0.08-0.2) |  |
|  |  |  | CC | 0 | 0 | 2 (2.94) |  |  |  |  |  |  |
| **9** | **IVS1+2016G>T^b^** | rs463599 | GG | 70 (100) | 54 (96.43) | 54 (80.6) |  | **G** | 1 | 0.98 (0.96-1.01) | 0.9 (0.85-0.95) |  |
|  |  |  | GT | 0 | 2 (3.57) | 13 (19.4) |  | **T** | 0 | 0.02 (-0.01-0.04) | 0.1 (0.05-0.15) |  |
|  |  |  | TT | 0 | 0 | 0 |  |  |  |  |  |  |
| **10** | **IVS1-1314G>A** | rs117910272 | GG | 68 (97.14) | 59 (98.33) | 61 (100) |  | **G** | 0.99 (0.97-1.01) | 0.99 (0.98-1.01) | 1 |  |
|  |  |  | GA | 2 (2.86) | 1 (1.67) | 0 |  | **A** | 0.01 (-0.01-0.03) | 0.01 (-0.01-0.02) | 0 |  |
|  |  |  | AA | 0 | 0 | 0 |  |  |  |  |  |  |
| **11** | **IVS1-613C>T** |  | CC | 68 (98.55) | 68 (100) | 70 (100) |  | **C** | 0.99 (0.98-1.01) | 1 | 1 |  |
|  |  |  | CT | 1 (1.45) | 0 | 0 |  | **T** | 0.01 (-0.01-0.02) | 0 | 0 |  |
|  |  |  | TT | 0 | 0 | 0 |  |  |  |  |  |  |
| **12** | **IVS1-207T>C^a^** | rs9457841 | TT | 16 (22.86) | 27 (39.13) | 27 (39.13) | 8 (21.1) | **T** | 0.46 (0.38-0.55) | 0.59 (0.51-0.68) | 0.59 (0.51-0.68) | 0.45 (0.34-0.56) |
|  |  |  | TC | 33 (47.14) | 28 (40.58) | 28 (40.58) | 18 (47.4) | **C** | 0.54 (0.45-0.62) | 0.41 (0.32-0.49) | 0.41 (0.32-0.49) | 0.55 (0.44-0.66) |
|  |  |  | CC | 21 (30) | 14 (20.29) | 14 (20.29) | 12 (31.6) |  |  |  |  |  |
| **13** | **IVS1-43T>G^a,b^** | rs4646272 | TT | 17 (24.29) | 27 (39.13) | 46 (66.67) | 10 (26.3) | **T** | 0.51 (0.42-0.59) | 0.62 (0.54-0.7) | 0.8 (0.73-0.86) | 0.49 (0.37-0.6) |
|  |  |  | TG | 37 (52.86) | 32 (46.38) | 18 (26.09) | 17 (44.7) | **G** | 0.49 (0.41-0.58) | 0.38 (0.3-0.46) | 0.2 (0.14-0.27) | 0.51 (0.4-0.63) |
|  |  |  | GG | 16 (22.86) | 10 (14.49) | 5 (7.25) | 11 (28.9) |  |  |  |  |  |
| **14** | **480C>G** | rs683369 | CC | 41 (58.57) | 45 (64.29) | 45 (64.29) |  | **C** | 0.79 (0.72-0.85) | 0.8 (0.73-0.87) | 0.79 (0.73-0.86) |  |
|  |  |  | CG | 28 (40) | 22 (31.43) | 21 (30) |  | **G** | 0.21 (0.15-0.28) | 0.2 (0.13-0.27) | 0.21 (0.14-0.27) |  |
|  |  |  | GG | 1 (1.43) | 3 (4.29) | 4 (5.71) |  |  |  |  |  |  |
| **15** | **494G>T^b^** |  | GG | 70 (100) | 69 (98.57) | 62 (88.57) |  | **G** | 1 | 0.99 (0.98-1.01) | 0.94 (0.9-0.98) |  |
|  |  |  | GT | 0 | 1 (1.43) | 8 (11.43) |  | **T** | 0 | 0.01 (-0.01-0.02) | 0.06 (0.02-0.1) |  |
|  |  |  | TT | 0 | 0 | 0 |  |  |  |  |  |  |
| **16** | **IVS2+97G>A^a,b^** | rs4646273 | GG | 22 (31.43) | 39 (56.52) | 59 (84.29) | 16 (42.1) | **G** | 0.61 (0.53-0.69) | 0.75 (0.67-0.82) | 0.91 (0.87-0.96) | 0.61 (0.5-0.72) |
|  |  |  | GA | 42 (60) | 25 (36.23) | 10 (14.29) | 14 (36.8) | **A** | 0.39 (0.31-0.47) | 0.25 (0.18-0.33) | 0.09 (0.04-0.13) | 0.39 (0.28-0.5) |
|  |  |  | AA | 6 (8.57) | 5 (7.25) | 1 (1.43) | 8 (21.1) |  |  |  |  |  |
| **17** | **IVS2+797C>G^a,b^** | rs4646274 | CC | 24 (34.29) | 32 (55.17) | 57 (83.82) | 16 (42.1) | **C** | 0.59 (0.51-0.67) | 0.74 (0.66-0.82) | 0.91 (0.86-0.96) | 0.61 (0.5-0.72) |
|  |  |  | CG | 35 (50) | 22 (37.93) | 10 (14.71) | 14 (36.8) | **G** | 0.41 (0.33-0.49) | 0.26 (0.18-0.34) | 0.09 (0.04-0.14) | 0.39 (0.28-0.5) |
|  |  |  | GG | 11 (15.71) | 4 (6.9) | 1 (1.47) | 8 (21.1) |  |  |  |  |  |
| **18** | **IVS2-1010T>G** | rs614890 | TT | 8 (11.43) | 7 (11.86) | 13 (19.4) |  | **T** | 0.3 (0.22-0.38) | 0.35 (0.26-0.43) | 0.38 (0.3-0.46) |  |
|  |  |  | TG | 26 (37.14) | 27 (45.76) | 25 (37.31) |  | **G** | 0.7 (0.62-0.78) | 0.65 (0.57-0.74) | 0.62 (0.54-0.7) |  |
|  |  |  | GG | 36 (51.43) | 25 (42.37) | 29 (43.28) |  |  |  |  |  |  |
| **19** | **IVS2-687G>T** | rs3798175 | GG | 56 (80) | 49 (81.67) | 56 (81.16) |  | **G** | 0.9 (0.85-0.95) | 0.91 (0.86-0.96) | 0.89 (0.84-0.94) |  |
|  |  |  | GT | 14 (20) | 11 (18.33) | 11 (15.94) |  | **T** | 0.1 (0.05-0.15) | 0.09 (0.04-0.14) | 0.11 (0.06-0.16) |  |
|  |  |  | TT | 0 | 0 | 2 (2.9) |  |  |  |  |  |  |
| **20** | **IVS2-461C>T** | rs3798174 | CC | 66 (94.29) | 55 (93.22) | 59 (84.29) |  | **C** | 0.97 (0.94-1) | 0.97 (0.93-1) | 0.91 (0.87-0.96) |  |
|  |  |  | CT | 4 (5.71) | 4 (6.78) | 10 (14.29) |  | **T** | 0.03 (0-0.06) | 0.03 (0-0.07) | 0.09 (0.04-0.13) |  |
|  |  |  | TT | 0 | 0 | 1 (1.43) |  |  |  |  |  |  |
| **21** | **IVS2-257C>T^a,b^** | rs4646275 | CC | 19 (27.14) | 31 (52.54) | 49 (70) | 16 (42.1) | **C** | 0.54 (0.46-0.63) | 0.7 (0.62-0.79) | 0.83 (0.77-0.89) | 0.59 (0.48-0.7) |
|  |  |  | CT | 38 (54.29) | 21 (35.59) | 18 (25.71) | 13 (34.2) | **T** | 0.46 (0.37-0.54) | 0.3 (0.21-0.38) | 0.17 (0.11-0.23) | 0.41 (0.3-0.52) |
|  |  |  | TT | 13 (18.57) | 7 (11.86) | 3 (4.29) | 9 (23.7) |  |  |  |  |  |
| **22** | **IVS2-99C>T** | rs3737088 | CC | 66 (94.29) | 57 (93.44) | 58 (84.06) |  | **C** | 0.97 (0.94-1) | 0.97 (0.94-1) | 0.91 (0.87-0.96) |  |
|  |  |  | CT | 4 (5.71) | 4 (6.56) | 10 (14.49) |  | **T** | 0.03 (0-0.06) | 0.03 (0-0.06) | 0.09 (0.04-0.13) |  |
|  |  |  | TT | 0 | 0 | 1 (1.45) |  |  |  |  |  |  |
| **23** | **IVS2-26C>T^b^** | rs45584532 | CC | 70 (100) | 59 (96.72) | 57 (81.43) |  | **C** | 1 | 0.98 (0.96-1.01) | 0.9 (0.85-0.95) |  |
|  |  |  | CT | 0 | 2 (3.28) | 12 (17.14) |  | **T** | 0 | 0.02 (-0.01-0.04) | 0.1 (0.05-0.15) |  |
|  |  |  | TT | 0 | 0 | 1 (1.43) |  |  |  |  |  |  |
| **24** | **IVS3+649-650insA^b^** | rs111438645 | wt | 70 (100) | 60 (96.77) | 56 (80) |  | **wt** | 1 | 0.98 (0.96-1.01) | 0.89 (0.84-0.94) |  |
|  |  |  | wt/ins | 0 | 2 (3.23) | 13 (18.57) |  | **ins** | 0 | 0.02 (-0.01-0.04) | 0.11 (0.06-0.16) |  |
|  |  |  | ins | 0 | 0 | 1 (1.43) |  |  |  |  |  |  |
| **25** | **IVS3-327C>T^b^** | rs3822840 | CC | 47 (72.31) | 40 (75.47) | 63 (100) |  | **C** | 0.86 (0.8-0.92) | 0.86 (0.79-0.92) | 1 |  |
|  |  |  | CT | 18 (27.69) | 11 (20.75) | 0 |  | **T** | 0.14 (0.08-0.2) | 0.14 (0.08-0.21) | 0 |  |
|  |  |  | TT | 0 | 2 (3.77) | 0 |  |  |  |  |  |  |
| **26** | **IVS4+597A>G^a^** | rs594709 | AA | 30 (52.63) | 21 (43.75) | 27 (43.55) | 24 (63.2) | **A** | 0.72 (0.64-0.8) | 0.68 (0.58-0.77) | 0.64 (0.55-0.72) | 0.76 (0.67-0.86) |
|  |  |  | AG | 22 (38.6) | 23 (47.92) | 25 (40.32) | 10 (26.3) | **G** | 0.28 (0.2-0.36) | 0.32 (0.23-0.42) | 0.36 (0.28-0.45) | 0.24 (0.14-0.33) |
|  |  |  | GG | 5 (8.77) | 4 (8.33) | 10 (16.13) | 4 (10.5) |  |  |  |  |  |
| **27** | **IVS4+865G>T^a,b^** | rs3798173 | GG | 16 (27.59) | 26 (50.98) | 41 (64.06) | 15 (39.5) | **G** | 0.52 (0.43-0.61) | 0.69 (0.6-0.78) | 0.8 (0.73-0.87) | 0.59 (0.48-0.7) |
|  |  |  | GT | 28 (48.28) | 18 (35.29) | 20 (31.25) | 15 (39.5) | **T** | 0.48 (0.39-0.57) | 0.31 (0.22-0.4) | 0.2 (0.13-0.27) | 0.41 (0.3-0.52) |
|  |  |  | TT | 14 (24.14) | 7 (13.73) | 3 (4.69) | 8 (21.1) |  |  |  |  |  |
| **28** | **IVS4+886C>G** | rs3798172 | CC | 58 (96.67) | 48 (96) | 55 (85.94) |  | **C** | 0.98 (0.96-1.01) | 0.98 (0.95-1.01) | 0.93 (0.89-0.97) |  |
|  |  |  | CG | 2 (3.33) | 2 (4) | 9 (14.06) |  | **G** | 0.02 (-0.01-0.04) | 0.02 (-0.01-0.05) | 0.07 (0.03-0.11) |  |
|  |  |  | GG | 0 | 0 | 0 |  |  |  |  |  |  |
| **29** | **IVS4+990C>T** | rs806383 | CC | 45 (70.31) | 26 (50.98) | 40 (63.49) |  | **C** | 0.84 (0.77-0.9) | 0.74 (0.65-0.82) | 0.77 (0.7-0.84) |  |
|  |  |  | CT | 17 (26.56) | 23 (45.1) | 17 (26.98) |  | **T** | 0.16 (0.1-0.23) | 0.26 (0.18-0.35) | 0.23 (0.16-0.3) |  |
|  |  |  | TT | 2 (3.13) | 2 (3.92) | 6 (9.52) |  |  |  |  |  |  |
| **30** | **IVS4+1028A>G** | rs3798170 | AA | 48 (76.19) | 42 (82.35) | 50 (80.65) |  | **A** | 0.88 (0.82-0.94) | 0.91 (0.86-0.97) | 0.9 (0.84-0.95) |  |
|  |  |  | AG | 15 (23.81) | 9 (17.65) | 11 (17.74) |  | **G** | 0.12 (0.06-0.18) | 0.09 (0.03-0.14) | 0.1 (0.05-0.16) |  |
|  |  |  | GG | 0 | 0 | 1 (1.61) |  |  |  |  |  |  |
| **31** | **IVS4+1040T>G^a,b^** | rs3798169 | TT | 18 (28.57) | 29 (56.86) | 52 (82.54) | 17 (44.7) | **T** | 0.56 (0.47-0.64) | 0.75 (0.66-0.83) | 0.9 (0.85-0.96) | 0.62 (0.51-0.73) |
|  |  |  | TG | 34 (53.97) | 18 (35.29) | 10 (15.87) | 13 (34.2) | **G** | 0.44 (0.36-0.53) | 0.25 (0.17-0.34) | 0.1 (0.04-0.15) | 0.38 (0.27-0.49) |
|  |  |  | GG | 11 (17.46) | 4 (7.84) | 1 (1.59) | 8 (21.1) |  |  |  |  |  |
| **32** | **IVS4-610C>T^b^** | rs3777392 | CC | 52 (76.47) | 50 (83.33) | 66 (97.06) |  | **C** | 0.88 (0.82-0.93) | 0.92 (0.87-0.97) | 0.99 (0.97-1.01) |  |
|  |  |  | CT | 15 (22.06) | 10 (16.67) | 2 (2.94) |  | **T** | 0.13 (0.07-0.18) | 0.08 (0.03-0.13) | 0.01 (-0.01-0.03) |  |
|  |  |  | TT | 1 (1.47) | 0 | 0 |  |  |  |  |  |  |
| **33** | **IVS4-98G>A^a,b^** | rs4646276 | GG | 19 (27.94) | 38 (63.33) | 48 (70.59) | 16 (42.1) | **G** | 0.55 (0.47-0.64) | 0.76 (0.68-0.83) | 0.84 (0.78-0.9) | 0.59 (0.48-0.7) |
|  |  |  | GA | 37 (54.41) | 15 (25) | 18 (26.47) | 13 (34.2) | **A** | 0.45 (0.36-0.53) | 0.24 (0.17-0.32) | 0.16 (0.1-0.22) | 0.41 (0.3-0.52) |
|  |  |  | AA | 12 (17.65) | 7 (11.67) | 2 (2.94) | 9 (23.7) |  |  |  |  |  |
| **34** | **IVS5-61G>A** | rs2282142 | GG | 52 (76.47) | 49 (80.33) | 57 (81.43) |  | **G** | 0.88 (0.83-0.94) | 0.9 (0.85-0.95) | 0.89 (0.84-0.94) |  |
|  |  |  | GA | 16 (23.53) | 12 (19.67) | 11 (15.71) |  | **A** | 0.12 (0.06-0.17) | 0.1 (0.05-0.15) | 0.11 (0.06-0.16) |  |
|  |  |  | AA | 0 | 0 | 2 (2.86) |  |  |  |  |  |  |
| **35** | **IVS5-7C>T^a,b^** | rs7762846 | CC | 68 (100) | 59 (96.72) | 56 (80) | 35 (94.6) | **C** | 1 | 0.98 (0.96-1.01) | 0.89 (0.84-0.94) | 0.97 (0.94-1.01) |
|  |  |  | CT | 0 | 2 (3.28) | 13 (18.57) | 2 (5.4) | **T** | 0 | 0.02 (-0.01-0.04) | 0.11 (0.06-0.16) | 0.03 (-0.01-0.06) |
|  |  |  | TT | 0 | 0 | 1 (1.43) | 0 (0) |  |  |  |  |  |
| **36** | **1022C>T** | rs2282143 | CC | 53 (76.8) | 52 (81.2) | 52 (80.9) |  | **C** | 0.88 (0.83-0.94) | 0.91 (0.86-0.96) | 0.89 (0.84-0.94) |  |
|  |  |  | CT | 16 (23.2) | 12 (18.8) | 12 (16.2) |  | **T** | 0.12 (0.06-0.17) | 0.09 (0.04-0.14) | 0.11 (0.06-0.16) |  |
|  |  |  | TT | 0 | 0 | 2 (2.9) |  |  |  |  |  |  |
| **37** | **IVS6+845C>T** | rs57874120 | CC | 62 (95.38) | 60 (93.75) | 58 (86.57) |  | **C** | 0.98 (0.95-1) | 0.97 (0.94-1) | 0.93 (0.88-0.97) |  |
|  |  |  | CT | 3 (4.62) | 4 (6.25) | 8 (11.94) |  | **T** | 0.02 (0-0.05) | 0.03 (0-0.06) | 0.07 (0.03-0.12) |  |
|  |  |  | TT | 0 | 0 | 1 (1.49) |  |  |  |  |  |  |
| **38** | **IVS6+1006G>A^a,b^** | rs4551194 | GG | 19 (29.23) | 34 (53.13) | 57 (83.82) | 16 (42.1) | **G** | 0.58 (0.49-0.66) | 0.73 (0.66-0.81) | 0.91 (0.86-0.96) | 0.61 (0.5-0.72) |
|  |  |  | GA | 37 (56.92) | 26 (40.63) | 10 (14.71) | 14 (36.8) | **A** | 0.42 (0.34-0.51) | 0.27 (0.19-0.34) | 0.09 (0.04-0.14) | 0.39 (0.28-0.5) |
|  |  |  | AA | 9 (13.85) | 4 (6.25) | 1 (1.47) | 8 (21.1) |  |  |  |  |  |
| **39** | **IVS6+1323G>A** | rs73025559 | GG | 61 (93.85) | 58 (93.55) | 58 (85.29) |  | **G** | 0.97 (0.94-1) | 0.97 (0.94-1) | 0.92 (0.87-0.96) |  |
|  |  |  | GA | 4 (6.15) | 4 (6.45) | 9 (13.24) |  | **A** | 0.03 (0-0.06) | 0.03 (0-0.06) | 0.08 (0.04-0.13) |  |
|  |  |  | AA | 0 | 0 | 1 (1.47) |  |  |  |  |  |  |
| **40** | **IVS6-1451T>G^a,b^** | rs1871388 | TT | 26 (40) | 36 (58.06) | 56 (82.35) | 14 (36.8) | **T** | 0.68 (0.6-0.76) | 0.76 (0.68-0.83) | 0.9 (0.85-0.95) | 0.58 (0.47-0.69) |
|  |  |  | TG | 37 (56.92) | 22 (35.48) | 11 (16.18) | 16 (42.1) | **G** | 0.32 (0.24-0.4) | 0.24 (0.17-0.32) | 0.1 (0.05-0.15) | 0.42 (0.31-0.53) |
|  |  |  | GG | 2 (3.08) | 4 (6.45) | 1 (1.47) | 8 (21.1) |  |  |  |  |  |
| **41** | **IVS6-14466-G>A** | rs73025560 | GG | 62 (95.38) | 60 (93.75) | 64 (94.12) |  | **G** | 0.98 (0.95-1) | 0.97 (0.94-1) | 0.96 (0.93-0.99) |  |
|  |  |  | GA | 3 (4.62) | 4 (6.25) | 3 (4.41) |  | **A** | 0.02 (0-0.05) | 0.03 (0-0.06) | 0.04 (0.01-0.07) |  |
|  |  |  | AA | 0 | 0 | 1 (1.47) |  |  |  |  |  |  |
| **42** | **IVS6-878C>A^ab^** | rs3798168 | CC | 19 (29.23) | 35 (56.45) | 58 (82.86) | 15 (39.5) | **C** | 0.58 (0.49-0.66) | 0.75 (0.67-0.83) | 0.91 (0.86-0.96) | 0.58 (0.47-0.69) |
|  |  |  | CA | 37 (56.92) | 23 (37.1) | 11 (15.71) | 14 (36.8) | **A** | 0.42 (0.34-0.51) | 0.25 (0.17-0.33) | 0.09 (0.04-0.14) | 0.42 (0.31-0.53) |
|  |  |  | AA | 9 (13.85) | 4 (6.45) | 1 (1.43) | 9 (23.7) |  |  |  |  |  |
| **43** | **IVS6-822G>A** |  | GG | 64 (100) | 61 (100) | 69 (98.57) |  | **G** | 1 | 1 | 0.99 (0.98-1.01) |  |
|  |  |  | GA | 0 | 0 | 1 (1.43) |  | **A** | 0 | 0 | 0.01 (-0.01-0.02) |  |
|  |  |  | AA | 0 | 0 | 0 |  |  |  |  |  |  |
| **44** | **1222A>G^a^** | rs628031 | AA | 7 (10.77) | 6 (9.68) | 13 (18.84) | 3 (7.9) | **A** | 0.27 (0.19-0.35) | 0.31 (0.23-0.4) | 0.42 (0.34-0.5) | 0.22 (0.13-0.32) |
|  |  |  | AG | 21 (32.31) | 27 (43.55) | 32 (46.38) | 11 (28.9) | **G** | 0.73 (0.65-0.81) | 0.69 (0.6-0.77) | 0.58 (0.5-0.66) | 0.78 (0.68-0.87) |
|  |  |  | GG | 37 (56.92) | 29 (46.77) | 24 (34.78) | 24 (63.2) |  |  |  |  |  |
| **45** | **1275insTGGTAAGT** | rs4646281 | wt | 39 (60) | 31 (49.21) | 32 (45.71) |  | **wt** | 0.75 (0.68-0.83) | 0.71 (0.63-0.79) | 0.68 (0.6-0.76) |  |
|  |  |  | wt/ins | 20 (30.77) | 27 (42.86) | 31 (44.29) |  | **ins** | 0.25 (0.17-0.32) | 0.29 (0.21-0.37) | 0.32 (0.24-0.4) |  |
|  |  |  | ins | 6 (9.23) | 5 (7.94) | 7 (10) |  |  |  |  |  |  |
| **46** | **IVS7+127T>C** |  | TT | 62 (100) | 60 (98.36) | 67 (100) |  | **T** | 1 | 0.99 (0.98-1.01) | 1 |  |
|  |  |  | TC | 0 | 1 (1.64) | 0 |  | **C** | 0 | 0.01 (-0.01-0.02) | 0 |  |
|  |  |  | CC | 0 | 0 | 0 |  |  |  |  |  |  |
| **47** | **IVS7+850C>T^ab^** |  | CC | 18 (28.13) | 34 (53.97) | 56 (82.35) | 15 (39.5) | **C** | 0.57 (0.48-0.66) | 0.73 (0.65-0.81) | 0.9 (0.85-0.95) | 0.59 (0.48-0.7) |
|  |  |  | CT | 37 (57.81) | 24 (38.1) | 11 (16.18) | 15 (39.5) | **T** | 0.43 (0.34-0.52) | 0.27 (0.19-0.35) | 0.1 (0.05-0.15) | 0.41 (0.3-0.52) |
|  |  |  | TT | 9 (14.06) | 5 (7.94) | 1 (1.47) | 8 (21.1) |  |  |  |  |  |
| **48** | **IVS7+1582G>A** | rs73025562 | GG | 48 (75) | 41 (65.08) | 40 (58.82) |  | **G** | 0.87 (0.81-0.93) | 0.82 (0.75-0.88) | 0.77 (0.7-0.84) |  |
|  |  |  | GA | 15 (23.44) | 21 (33.33) | 25 (36.76) |  | **A** | 0.13 (0.07-0.19) | 0.18 (0.12-0.25) | 0.23 (0.16-0.3) |  |
|  |  |  | AA | 1 (1.56) | 1 (1.59) | 3 (4.41) |  |  |  |  |  |  |
| **49** | **IVS7+1624A>T** | rs73784003 | AA | 62 (96.88) | 59 (93.65) | 59 (86.76) |  | **A** | 0.98 (0.96-1.01) | 0.97 (0.94-1) | 0.93 (0.88-0.97) |  |
|  |  |  | AT | 2 (3.13) | 4 (6.35) | 8 (11.76) |  | **T** | 0.02 (-0.01-0.04) | 0.03 (0-0.06) | 0.07 (0.03-0.12) |  |
|  |  |  | TT | 0 | 0 | 1 (1.47) |  |  |  |  |  |  |
| **50** | **IVS7+1660G>A** |  | GG | 64 (100) | 63 (100) | 65 (95.59) |  | **G** | 1 | 1 | 0.98 (0.95-1) |  |
|  |  |  | GA | 0 | 0 | 3 (4.41) |  | **A** | 0 | 0 | 0.02 (0-0.05) |  |
|  |  |  | AA | 0 | 0 | 0 |  |  |  |  |  |  |
| **51** | **IVS7-1368C>T^b^** | rs1867350 | CC | 20 (28.99) | 36 (57.14) | 55 (84.62) |  | **C** | 0.57 (0.48-0.65) | 0.75 (0.68-0.83) | 0.92 (0.87-0.96) |  |
|  |  |  | CT | 38 (55.07) | 23 (36.51) | 9 (13.85) |  | **T** | 0.43 (0.35-0.52) | 0.25 (0.17-0.32) | 0.08 (0.04-0.13) |  |
|  |  |  | TT | 11 (15.94) | 4 (6.35) | 1 (1.54) |  |  |  |  |  |  |
| **52** | **IVS7-1053C>T** | rs7766568 | CC | 66 (95.65) | 56 (93.33) | 52 (83.87) |  | **C** | 0.98 (0.95-1) | 0.97 (0.93-1) | 0.91 (0.86-0.96) |  |
|  |  |  | CT | 3 (4.35) | 4 (6.67) | 9 (14.52) |  | **T** | 0.02 (0-0.05) | 0.03 (0-0.07) | 0.09 (0.04-0.14) |  |
|  |  |  | TT | 0 | 0 | 1 (1.61) |  |  |  |  |  |  |
| **53** | **IVS7-988A>T** |  | AA | 69 (100) | 60 (98.36) | 61 (100) |  | **A** | 1 | 0.99 (0.98-1.01) | 1 |  |
|  |  |  | AT | 0 | 1 (1.64) | 0 |  | **T** | 0 | 0.01 (-0.01-0.02) | 0 |  |
|  |  |  | TT | 0 | 0 | 0 |  |  |  |  |  |  |
| **54** | **IVS8+523G>A** |  | GG | 68 (97.14) | 60 (98.36) | 68 (100) |  | **G** | 0.99 (0.97-1.01) | 0.99 (0.98-1.01) | 1 |  |
|  |  |  | GA | 2 (2.86) | 1 (1.64) | 0 |  | **A** | 0.01 (-0.01-0.03) | 0.01 (-0.01-0.02) | 0 |  |
|  |  |  | AA | 0 | 0 | 0 |  |  |  |  |  |  |
| **55** | **IVS8+1322G>T^a^** | rs644992 | GG | 31 (46.97) | 18 (31.58) | 24 (35.29) | 19 (50) | **G** | 0.71 (0.63-0.79) | 0.58 (0.49-0.67) | 0.6 (0.52-0.69) | 0.68 (0.58-0.79) |
|  |  |  | GT | 32 (48.48) | 30 (52.63) | 34 (50) | 14 (36.8) | **T** | 0.29 (0.21-0.37) | 0.42 (0.33-0.51) | 0.4 (0.31-0.48) | 0.32 (0.21-0.42) |
|  |  |  | TT | 3 (4.55) | 9 (15.79) | 10 (14.71) | 5 (13.2) |  |  |  |  |  |
| **56** | **IVS8+1711T>A^b^** |  | TT | 52 (78.79) | 49 (84.48) | 46 (65.71) |  | **T** | 0.89 (0.84-0.95) | 0.9 (0.84-0.95) | 0.82 (0.76-0.88) |  |
|  |  |  | TA | 14 (21.21) | 6 (10.34) | 23 (32.86) |  | **A** | 0.11 (0.05-0.16) | 0.1 (0.05-0.16) | 0.18 (0.12-0.24) |  |
|  |  |  | AA | 0 | 3 (5.17) | 1 (1.43) |  |  |  |  |  |  |
| **57** | **IVS8+1780T>A** |  | TT | 65 (97.01) | 59 (100) | 70 (100) |  | **T** | 0.99 (0.96-1.01) | 1 | 1 |  |
|  |  |  | TA | 2 (2.99) | 0 | 0 |  | **A** | 0.01 (-0.01-0.04) | 0 | 0 |  |
|  |  |  | AA | 0 | 0 | 0 |  |  |  |  |  |  |
| **58** | **IVS8+2228C>T** |  | CC | 66 (100) | 62 (100) | 69 (98.57) |  | **C** | 1 | 1 | 0.99 (0.98-1.01) |  |
|  |  |  | CT | 0 | 0 | 1 (1.43) |  | **T** | 0 | 0 | 0.01 (-0.01-0.02) |  |
|  |  |  | TT | 0 | 0 | 0 |  |  |  |  |  |  |
| **59** | **IVS8+2698A>G** | rs637841 | AA | 32 (50) | 19 (32.76) | 26 (37.68) |  | **A** | 0.73 (0.65-0.8) | 0.58 (0.49-0.67) | 0.61 (0.53-0.69) |  |
|  |  |  | AG | 29 (45.31) | 29 (50) | 32 (46.38) |  | **G** | 0.27 (0.2-0.35) | 0.42 (0.33-0.51) | 0.39 (0.31-0.47) |  |
|  |  |  | GG | 3 (4.69) | 10 (17.24) | 11 (15.94) |  |  |  |  |  |  |
| **60** | **IVS8+3668G>A** |  | GG | 70 (100) | 53 (100) | 61 (98.39) |  | **G** | 1 | 1 | 0.99 (0.98-1.01) |  |
|  |  |  | GA | 0 | 0 | 1 (1.61) |  | **A** | 0 | 0 | 0.01 (-0.01-0.02) |  |
|  |  |  | AA | 0 | 0 | 0 |  |  |  |  |  |  |
| **61** | **IVS8+3722A>G** |  | AA | 69 (98.57) | 51 (96.23) | 55 (88.71) |  | **A** | 0.99 (0.98-1.01) | 0.97 (0.94-1) | 0.94 (0.89-0.98) |  |
|  |  |  | AG | 1 (1.43) | 1 (1.89) | 6 (9.68) |  | **G** | 0.01 (-0.01-0.02) | 0.03 (0-0.06) | 0.06 (0.02-0.11) |  |
|  |  |  | GG | 0 | 1 (1.89) | 1 (1.61) |  |  |  |  |  |  |
| **62** | **IVS8+4215T>C** | rs654993 | TT | 9 (13.43) | 17 (26.56) | 11 (15.71) |  | **T** | 0.44 (0.36-0.52) | 0.48 (0.4-0.57) | 0.42 (0.34-0.5) |  |
|  |  |  | TC | 41 (61.19) | 28 (43.75) | 37 (52.86) |  | **C** | 0.56 (0.48-0.64) | 0.52 (0.43-0.6) | 0.58 (0.5-0.66) |  |
|  |  |  | CC | 17 (25.37) | 19 (29.69) | 22 (31.43) |  |  |  |  |  |  |
| **63** | **IVS8+4316G>A** | rs2197296 | GG | 34 (50.75) | 30 (46.15) | 46 (65.71) |  | **G** | 0.73 (0.66-0.81) | 0.67 (0.59-0.75) | 0.79 (0.72-0.85) |  |
|  |  |  | GA | 30 (44.78) | 27 (41.54) | 18 (25.71) |  | **A** | 0.27 (0.19-0.34) | 0.33 (0.25-0.41) | 0.21 (0.15-0.28) |  |
|  |  |  | AA | 3 (4.48) | 8 (12.31) | 6 (8.57) |  |  |  |  |  |  |
| **64** | **IVS8+4387C>T^b^** | rs62440901 | CC | 68 (100) | 60 (92.31) | 52 (74.29) |  | **C** | 1 | 0.96 (0.93-0.99) | 0.87 (0.82-0.93) |  |
|  |  |  | CT | 0 | 5 (7.69) | 18 (25.71) |  | **T** | 0 | 0.04 (0.01-0.07) | 0.13 (0.07-0.18) |  |
|  |  |  | TT | 0 | 0 | 0 |  |  |  |  |  |  |
| **65** | **IVS8+5067G>T** |  | GG | 67 (98.53) | 56 (100) | 69 (100) |  | **G** | 0.99 (0.98-1.01) | 1 | 1 |  |
|  |  |  | GT | 1 (1.47) | 0 | 0 |  | **T** | 0.01 (-0.01-0.02) | 0 | 0 |  |
|  |  |  | TT | 0 | 0 | 0 |  |  |  |  |  |  |
| **66** | **IVS8+5101T>C** | rs7750592 | TT | 35 (51.47) | 24 (42.86) | 32 (46.38) |  | **T** | 0.74 (0.67-0.82) | 0.63 (0.54-0.71) | 0.68 (0.6-0.76) |  |
|  |  |  | TC | 31 (45.59) | 22 (39.29) | 30 (43.48) |  | **C** | 0.26 (0.18-0.33) | 0.38 (0.29-0.46) | 0.32 (0.24-0.4) |  |
|  |  |  | CC | 2 (2.94) | 10 (17.86) | 7 (10.14) |  |  |  |  |  |  |
| **67** | **IVS8+5402T>C^a^** |  | TT | 2 (3.08) | 4 (7.02) | 9 (13.85) | 1 (2.7) | **T** | 0.17 (0.1-0.23) | 0.21 (0.14-0.29) | 0.32 (0.24-0.4) | 0.15 (0.07-0.23) |
|  |  |  | TC | 18 (27.69) | 16 (28.07) | 24 (36.92) | 9 (24.3) | **C** | 0.83 (0.77-0.9) | 0.79 (0.71-0.86) | 0.68 (0.6-0.76) | 0.85 (0.77-0.93) |
|  |  |  | CC | 45 (69.23) | 37 (64.91) | 32 (49.23) | 27 (73) |  |  |  |  |  |
| **68** | **IVS8+5491A>G** | rs9295124 | AA | 34 (53.13) | 25 (43.86) | 33 (51.56) |  | **A** | 0.75 (0.67-0.83) | 0.62 (0.53-0.71) | 0.71 (0.63-0.79) |  |
|  |  |  | AG | 28 (43.75) | 21 (36.84) | 25 (39.06) |  | **G** | 0.25 (0.17-0.33) | 0.38 (0.29-0.47) | 0.29 (0.21-0.37) |  |
|  |  |  | GG | 2 (3.13) | 11 (19.3) | 6 (9.38) |  |  |  |  |  |  |
| **69** | **IVS8-5206C>T^a^** | rs13212914 | CC | 35 (50) | 26 (43.33) | 35 (50) | 13 (34.2) | **C** | 0.74 (0.66-0.81) | 0.63 (0.55-0.72) | 0.69 (0.62-0.77) | 0.58 (0.47-0.69) |
|  |  |  | CT | 33 (47.14) | 24 (40) | 27 (38.57) | 18 (47.4) | **T** | 0.26 (0.19-0.34) | 0.37 (0.28-0.45) | 0.31 (0.23-0.38) | 0.42 (0.31-0.53) |
|  |  |  | TT | 2 (2.86) | 10 (16.67) | 8 (11.43) | 7 (18.4) |  |  |  |  |  |
| **70** | **IVS8-5061delAGTA** |  | wt | 63 (90) | 59 (98.33) | 69 (98.57) |  | **wt** | 0.95 (0.91-0.99) | 0.99 (0.98-1.01) | 0.99 (0.98-1.01) |  |
|  |  |  | wt/del | 7 (10) | 1 (1.67) | 1 (1.43) |  | **del** | 0.05 (0.01-0.09) | 0.01 (-0.01-0.02) | 0.01 (-0.01-0.02) |  |
|  |  |  | del | 0 | 0 | 0 |  |  |  |  |  |  |
| **71** | **IVS8-5054C>T** |  | CC | 65 (92.86) | 59 (98.33) | 70 (100) |  | **C** | 0.96 (0.93-1) | 0.98 (0.96-1.01) | 1 |  |
|  |  |  | CT | 5 (7.14) | 0 | 0 |  | **T** | 0.04 (0-0.07) | 0.02 (-0.01-0.04) | 0 |  |
|  |  |  | TT | 0 | 1 (1.67) | 0 |  |  |  |  |  |  |
| **72** | **IVS8-4331A>G^a^** | rs9347386 | AA | 32 (49.23) | 25 (44.64) | 35 (50) | 15 (39.5) | **A** | 0.74 (0.66-0.81) | 0.64 (0.55-0.73) | 0.69 (0.61-0.76) | 0.59 (0.48-0.7) |
|  |  |  | AG | 32 (49.23) | 22 (39.29) | 26 (37.14) | 15 (39.5) | **G** | 0.26 (0.19-0.34) | 0.36 (0.27-0.45) | 0.31 (0.24-0.39) | 0.41 (0.3-0.52) |
|  |  |  | GG | 1 (1.54) | 9 (16.07) | 9 (12.86) | 8 (21.1) |  |  |  |  |  |
| **73** | **IVS8-3776T>C** | rs73025574 | TT | 63 (95.45) | 52 (91.23) | 61 (88.41) |  | **T** | 0.98 (0.95-1) | 0.96 (0.92-0.99) | 0.93 (0.89-0.98) |  |
|  |  |  | TC | 3 (4.55) | 5 (8.77) | 7 (10.14) |  | **C** | 0.02 (0-0.05) | 0.04 (0.01-0.08) | 0.07 (0.02-0.11) |  |
|  |  |  | CC | 0 | 0 | 1 (1.45) |  |  |  |  |  |  |
| **74** | **IVS8-3551C>T** | rs619598 | CC | 66 (100) | 53 (94.64) | 60 (86.96) |  | **C** | 1 | 0.97 (0.94-1) | 0.91 (0.87-0.96) |  |
|  |  |  | CT | 0 | 3 (5.36) | 6 (8.7) |  | **T** | 0 | 0.03 (0-0.06) | 0.09 (0.04-0.13) |  |
|  |  |  | TT | 0 | 0 | 3 (4.35) |  |  |  |  |  |  |
| **75** | **IVS8-3273T>A** | rs9347388 | TT | 33 (48.53) | 22 (39.29) | 33 (49.25) |  | **T** | 0.73 (0.65-0.8) | 0.59 (0.5-0.68) | 0.69 (0.61-0.77) |  |
|  |  |  | TA | 33 (48.53) | 22 (39.29) | 26 (.) |  | **A** | 0.27 (0.2-0.35) | 0.41 (0.32-0.5) | 0.31 (0.23-0.39) |  |
|  |  |  | AA | 2 (2.94) | 12 (21.43) | 8 (.) |  |  |  |  |  |  |
| **76** | **IVS8-2964C>A^b^** | rs622342 | CC | 2 (3.08) | 3 (5.45) | 9 (14.29) |  | **C** | 0.15 (0.09-0.22) | 0.2 (0.13-0.27) | 0.37 (0.28-0.45) |  |
|  |  |  | CA | 16 (24.62) | 16 (29.09) | 28 (44.44) |  | **A** | 0.85 (0.78-0.91) | 0.8 (0.73-0.87) | 0.63 (0.55-0.72) |  |
|  |  |  | AA | 47 (72.31) | 36 (65.45) | 26 (41.27) |  |  |  |  |  |  |
| **77** | **IVS8-2423C>T** | rs1382785 | CC | 34 (50) | 22 (37.29) | 36 (52.17) |  | **C** | 0.69 (0.61-0.77) | 0.64 (0.55-0.72) | 0.71 (0.63-0.79) |  |
|  |  |  | CT | 26 (38.24) | 31 (52.54) | 26 (37.68) |  | **T** | 0.31 (0.23-0.39) | 0.36 (0.28-0.45) | 0.29 (0.21-0.37) |  |
|  |  |  | TT | 8 (11.76) | 6 (10.17) | 7 (10.14) |  |  |  |  |  |  |
| **78** | **IVS8-1803C>T^a^** | rs4709401 | CC | 35 (50.72) | 28 (42.42) | 32 (46.38) | 14 (36.8) | **C** | 0.72 (0.65-0.8) | 0.63 (0.55-0.71) | 0.68 (0.6-0.76) | 0.58 (0.47-0.69) |
|  |  |  | CT | 30 (43.48) | 27 (40.91) | 30 (43.48) | 16 (42.1) | **T** | 0.28 (0.2-0.35) | 0.37 (0.29-0.45) | 0.32 (0.24-0.4) | 0.42 (0.31-0.53) |
|  |  |  | TT | 4 (5.8) | 11 (16.67) | 7 (10.14) | 8 (21.1) |  |  |  |  |  |
| **79** | **IVS8-1793C>T** | rs4709402 | CC | 37 (53.62) | 28 (42.42) | 32 (46.38) |  | **C** | 0.74 (0.67-0.81) | 0.62 (0.54-0.7) | 0.68 (0.6-0.76) |  |
|  |  |  | CT | 28 (40.58) | 26 (39.39) | 30 (43.48) |  | **T** | 0.26 (0.19-0.33) | 0.38 (0.3-0.46) | 0.32 (0.24-0.4) |  |
|  |  |  | TT | 4 (5.8) | 12 (18.18) | 7 (10.14) |  |  |  |  |  |  |
| **80** | **IVS8-1295C>A** | rs650284 | CC | 1 (1.47) | 4 (5.97) | 9 (13.04) |  | **C** | 0.15 (0.09-0.22) | 0.19 (0.13-0.26) | 0.32 (0.24-0.4) |  |
|  |  |  | CA | 19 (27.94) | 18 (26.87) | 26 (37.68) |  | **A** | 0.85 (0.78-0.91) | 0.81 (0.74-0.87) | 0.68 (0.6-0.76) |  |
|  |  |  | AA | 48 (70.59) | 45 (67.16) | 34 (49.28) |  |  |  |  |  |  |
| **81** | **IVS8-926A>T** |  | AA | 66 (95.65) | 65 (100) | 69 (100) |  | **A** | 0.98 (0.95-1) | 1 | 1 |  |
|  |  |  | AT | 3 (4.35) | 0 | 0 |  | **T** | 0.02 (0-0.05) | 0 | 0 |  |
|  |  |  | TT | 0 | 0 | 0 |  |  |  |  |  |  |
| **82** | **IVS8-464G>A^b^** | rs11753995 | GG | 69 (100) | 59 (93.65) | 48 (70.59) |  | **G** | 1 | 0.96 (0.93-0.99) | 0.8 (0.73-0.87) |  |
|  |  |  | GA | 0 | 3 (4.76) | 13 (19.12) |  | **A** | 0 | 0.04 (0.01-0.07) | 0.2 (0.13-0.27) |  |
|  |  |  | AA | 0 | 1 (1.59) | 7 (10.29) |  |  |  |  |  |  |
| **83** | **IVS9+43C>T** | rs2297374 | CC | 35 (53.03) | 27 (40.91) | 32 (45.71) |  | **C** | 0.73 (0.66-0.81) | 0.61 (0.53-0.7) | 0.67 (0.59-0.75) |  |
|  |  |  | CT | 27 (40.91) | 27 (40.91) | 30 (42.86) |  | **T** | 0.27 (0.19-0.34) | 0.39 (0.3-0.47) | 0.33 (0.25-0.41) |  |
|  |  |  | TT | 4 (6.06) | 12 (18.18) | 8 (11.43) |  |  |  |  |  |  |
| **84** | **IVS9-38G>A** | rs41267795 | GG | 68 (100) | 66 (97.06) | 68 (98.55) |  | **G** | 1 | 0.99 (0.97-1.01) | 0.99 (0.98-1.01) |  |
|  |  |  | GA | 0 | 2 (2.94) | 1 (1.45) |  | **A** | 0 | 0.01 (-0.01-0.03) | 0.01 (-0.01-0.02) |  |
|  |  |  | AA | 0 | 0 | 0 |  |  |  |  |  |  |
| **85** | **1503G>A** | rs41267797 | GG | 68 (100) | 65 (95.59) | 66 (95.65) |  | **G** | 1 | 0.98 (0.95-1) | 0.98 (0.95-1) |  |
|  |  |  | GA | 0 | 3 (4.41) | 3 (4.35) |  | **A** | 0 | 0.02 (0-0.05) | 0.02 (0-0.05) |  |
|  |  |  | AA | 0 | 0 | 0 |  |  |  |  |  |  |
| **86** | **IVS10+393G>C** | rs3818678 | GG | 32 (47.06) | 26 (38.81) | 31 (44.93) |  | **G** | 0.71 (0.64-0.79) | 0.61 (0.53-0.69) | 0.67 (0.59-0.75) |  |
|  |  |  | GC | 33 (48.53) | 30 (44.78) | 30 (43.48) |  | **C** | 0.29 (0.21-0.36) | 0.39 (0.31-0.47) | 0.33 (0.25-0.41) |  |
|  |  |  | CC | 3 (4.41) | 11 (16.42) | 8 (11.59) |  |  |  |  |  |  |
| **87** | **IVS10-688T>C^ab^** | rs1564348 | TT | 59 (100) | 47 (92.16) | 52 (76.47) | 38 (100) | **T** | 1 | 0.96 (0.92-1) | 0.88 (0.83-0.94) | 1 |
|  |  |  | TC | 0 | 4 (7.84) | 16 (23.53) | 0 | **C** | 0 | 0.04 (0-0.08) | 0.12 (0.06-0.17) | 0 |
|  |  |  | CC | 0 | 0 | 0 | 0 |  |  |  |  |  |
| **88** | **IVS10-6349C>T^b^** | rs609468 | CC | 7 (13.21) | 21 (46.67) | 27 (40.91) |  | **C** | 0.41 (0.31-0.5) | 0.67 (0.57-0.76) | 0.65 (0.57-0.73) |  |
|  |  |  | CT | 29 (54.72) | 18 (40) | 32 (48.48) |  | **T** | 0.59 (0.5-0.69) | 0.33 (0.24-0.43) | 0.35 (0.27-0.43) |  |
|  |  |  | TT | 17 (32.08) | 6 (13.33) | 7 (10.61) |  |  |  |  |  |  |
| **89** | **IVS10-21C>T^b^** | rs622591 | CC | 9 (13.43) | 20 (32.79) | 27 (38.57) |  | **C** | 0.4 (0.31-0.48) | 0.56 (0.47-0.65) | 0.64 (0.56-0.72) |  |
|  |  |  | CT | 35 (52.24) | 28 (45.9) | 35 (50) |  | **T** | 0.6 (0.52-0.69) | 0.44 (0.35-0.53) | 0.36 (0.28-0.44) |  |
|  |  |  | TT | 23 (34.33) | 13 (21.31) | 8 (11.43) |  |  |  |  |  |  |
|  |  |  |  |  |  |  |  |  |  |  |  |  |
|  | ***ABCB1^c^*** |  |  |  |  |  |  |  |  |  |  |  |
| **90** | **1236C>T** | rs1128503 | CC | 50 (52.1) | 40 (43.5) | 42 (48.3) | 6 (15.8) | **C** | 28.1 (21.6–34.6) | 34.2 (27.3–41.1) | 32.8 (25.4–40.2) | 0.3 (0.2-0.41) |
|  |  |  | CT | 38 (39.6) | 41 (44.6) | 33 (37.9) | 11 (28.9) | **T** | 71.9 (65.6–78.4) | 65.8 (58.9–72.7) | 67.2 (59.8–74.6) | 0.7 (0.59-0.8) |
|  |  |  | TT | 8 (8.3) | 11 (12) | 12 (13.8) | 21 (55.3) |  |  |  |  |  |
| **91** | **2677G>T/A** | rs2032582 | AA | 1 (1) | 0 | 0 | 8 (21.1) | **G** | 37.5 (30.2–44.8) | 52.7 (45.4–60.0) | 33.3 (25.9–40.7) | 0.47 (0.36-0.59) |
|  |  |  | AG | 8 (8.4) | 2 (2.2) | 7 (8.1) | 14 (36.8) | **T** | 50.0 (42.7–57.3) | 44.0 (37.0–51.0) | 59.8 (51.7–67.9) | 0.45 (0.34-0.56) |
|  |  |  | AT | 14 (14.6) | 4 (4.4) | 5 (5.8) | 10 (26.3) | **A** | 12.5 (8.0–17.0 | 3.3 (0.7–5.9) | 6.9 (3.3–10.5) | 0.08 (0.02-0.14) |
|  |  |  | GG | 16 (16.7) | 26 (28.3) | 12 (13.8) | 6 (15.8) |  |  |  |  |  |
|  |  |  | GT | 32 (33.3) | 43 (46.6) | 27 (31) |  |  |  |  |  |  |
|  |  |  | TT | 25 (26) | 17 (18.5) | 36 (41.4) |  |  |  |  |  |  |
| **92** | **3435C>T** | rs1045642 | CC | 24 (25) | 25 (27.2) | 16 (18.4) | 13 (34.2) | **C** | 46.9 (39.5–54.3) | 48.9 (41.2–56.6) | 36.8 (28.9–44.7) | 0.54 (0.43-0.65) |
|  |  |  | CT | 42 (43.7) | 40 (43.5) | 32 (36.8) | 15 (39.5) | **T** | 53.1 (45.7–60.5) | 51.1 (43.4–58.8) | 63.2 (55.3–71.1 | 0.46 (0.35-0.57) |
|  |  |  | TT | 30 (31.3) | 27 (29.3) | 39 (44.8) | 10 (26.3) |  |  |  |  |  |
|  |  |  |  |  |  |  |  |  |  |  |  |  |
|  | ***ABCG2^c^*** |  |  |  |  |  |  |  |  |  |  |  |
| **93** | **421C>A** | rs2231142 | CC | 49 (52.1) | 54 (55.7) | 69 (73.4) | 19 (67.9) | **C** | 0.72 (0.66-0.79) | 0.73 (0.66-0.79) | 0.85 (0.79-0.90) | 0.8 (0.7-0.91) |
|  |  |  | CA | 38 (40.4) | 33 (34) | 21 (22.3) | 7 (25) | **A** | 0.28 (0.21-0.34) | 0.27 (0.21-0.34) | 0.15 (0.10-0.21) | 0.2 (0.09-0.3) |
|  |  |  | AA | 7 (7.4) | 10 (10.3) | 4 (4.3) | 2 (7.1) |  |  |  |  |  |
|  |  |  |  |  |  |  |  |  |  |  |  |  |
|  | ***CYP3A5*3^c^*** |  |  |  |  |  |  |  |  |  |  |  |
| **94** | **22893A>G** | rs776746 | AA | 9 (8.3) | 10 (10.2) | 11 (12.2) | 2 (5.3) | **A** | 0.25 (0.18–0.31) | 0.39 (0.33–0.45) | 0.41 (0.34–0.47) | 0.29 (0.19-0.39) |
|  |  |  | AG | 35 (32.4) | 56 (57.1) | 51 (56.7) | 18 (47.4) | **G** | 0.76 (0.69–0.82) | 0.61 (0.55–0.67) | 0.59 (0.53–0.66) | 0.71 (0.61-0.81) |
|  |  |  | GG | 64 (59.3) | 32 (32.7) | 28 (31.1) | 18 (47.4) |  |  |  |  |  |
|  |  |  |  |  |  |  |  |  |  |  |  |  |
|  | ***PXR^c^*** |  |  |  |  |  |  |  |  |  |  |  |
| **95** | **IVS2+55A>G** | rs1464603 | AA | 34 (34) | 26 (26) | 42 (42) | 17 (44.7) | **A** | 0.59 (0.52-0.66) | 0.51 (0.44-0.58) | 0.62 (0.55-0.69) | 0.66 (0.55-0.76) |
|  |  |  | AG | 50 (50) | 50 (50) | 40 (40) | 16 (42.1) | **G** | 0.41 (0.34-0.48) | 0.49 (0.42-0.56) | 0.38 (0.31-0.45) | 0.34 (0.24-0.45) |
|  |  |  | GG | 16 (16) | 24 (24) | 18 (18) | 5 (13.2) |  |  |  |  |  |
| **96** | **IVS2+78A>G** | rs1464602 | AA | 34 (34) | 30 (30) | 42 (42) | 17 (44.7) | **A** | 0.6 (0.53-0.67) | 0.54 (0.46-0.61) | 0.63 (0.55-0.70) | 0.66 (0.55-0.76) |
|  |  |  | AG | 52 (52) | 47 (47) | 41 (41) | 16 (42.1) | **G** | 0.4 (0.33-0.47) | 0.46 (0.39-0.54) | 0.37 (0.30-0.45) | 0.34 (0.24-0.45) |
|  |  |  | GG | 14 (14) | 23 (23) | 17 (17) | 5 (13.2) |  |  |  |  |  |
| **97** | **IVS6-17C>T** | rs2276707 | CC | 31 (31) | 33 (33) | 58 (58) | 12 (31.6) | **C** | 0.55 (0.47-0.62) | 0.57 (0.49-0.64) | 0.76 (0.69-0.82) | 0.53 (0.41-0.64) |
|  |  |  | CT | 47 (47) | 47 (47) | 35 (35) | 16 (42.1) | **T** | 0.45 (0.38-0.53) | 0.43 (0.36-0.51) | 0.24 (0.18-0.31) | 0.47 (0.36-0.59) |
|  |  |  | TT | 22 (22) | 20 (20) | 7 (7) | 10 (26.3) |  |  |  |  |  |
| **98** | **1792A>G** | rs3732359 | AA | 34 (34) | 46 (46) | 21 (21) | 8 (21.1) | **A** | 0.57 (0.50-0.64) | 0.66 (0.59-0.73) | 0.41 (0.33-0.48) | 0.41 (0.3-0.52) |
|  |  |  | AG | 46 (46) | 40 (40) | 39 (39) | 15 (39.5) | **G** | 0.43 (0.36-0.50) | 0.34 (0.27-0.41) | 0.59 (0.52-0.67) | 0.59 (0.48-0.7) |
|  |  |  | GG | 20 (20) | 14 (14) | 40 (40) | 15 (39.5) |  |  |  |  |  |
| **99** | **1944T>C** | rs3732360 | TT | 34 (34) | 47 (47) | 21 (21) | 8 (21.1) | **T** | 0.57 (0.50-0.64) | 0.67 (0.59-0.74) | 0.41 (0.33-0.48) | 0.42 (0.31-0.53) |
|  |  |  | TC | 46 (46) | 39 (39) | 39 (39) | 16 (42.1) | **C** | 0.43 (0.36-0.50) | 0.33 (0.26-0.41) | 0.59 (0.52-0.67) | 0.58 (0.47-0.69) |
|  |  |  | CC | 20 (20) | 14 (14) | 40 (40) | 14 (36.8) |  |  |  |  |  |
|  |  |  |  |  |  |  |  |  |  |  |  |  |
| **100** | **2654T>C** | rs3814058 | TT | 29 (29) | 37 (37) | 59 (59) | 12 (32.4) | **T** | 0.55 (0.48-0.61) | 0.58 (0.51-0.65) | 0.76 (0.70-0.82) | 0.54 (0.43-0.65) |
|  |  |  | TC | 51 (51) | 42 (42) | 34 (34) | 16 (43.2) | **C** | 0.46 (0.39-0.52) | 0.42 (0.35-0.49) | 0.24 (0.18-0.30) | 0.46 (0.35-0.57) |
|  |  |  | CC | 20 (20) | 21 (21) | 7 (7) | 9 (24.3) |  |  |  |  |  |

^a^ Tag-SNPs

^b^ SNPs showed statistically significant interethnic differences in the genotypic distributions among healthy subjects of three ethnic groups Chinese, Malay and Indians (X^2^ test, p<0.016)

^c^ Healthy data for *ABCB1, ABCG2, CYP3A5* and *PXR* is taken from our previous publications [30-33]
