# Supplementary material for: SLC22A1-ABCB1 Haplotype Profiles Predict Imatinib Pharmacokinetics in Asian Patients with Chronic Myeloid Leukemia
Source: PLoS One. 2012 Dec 18;7(12):e51771. doi: 10.1371/journal.pone.0051771 (PMC3525665; doi:10.1371/journal.pone.0051771)
Supplement: Table S2 — Primer Sequences and PCR Conditions for Amplifications of SLC22A1 regions (UCSC RefSeq: NM_003057). (DOC) [file pone.0051771.s004.doc]

**Table S2.** Primer Sequences and PCR Conditions for Amplifications of *SLC22A1* regions (UCSC RefSeq: NM_003057).

| **SN** | **Sequence Coverage** | **Primer Sequences** | **Fragment Sizes (bp)** | **Optimum Annealing Temperature (°C)** | **Number of Amplification Cycles** |
| --- | --- | --- | --- | --- | --- |
| **1** | 5’ Upstream | F: 5′-ACACAAACTCAGGCATCAGAAAT-3′ | 902 | 55.9 | 32 |
|  |  | R: 5′-TTTGGTTTTCCATAAGTTGAACA-3′ |  |  |  |
| **2** | 5’ Upstream | F: 5′-ATCTTGTTCAAGTGAAAGCCAAA-3′ | 1177 | 64 | 35 |
|  |  | R: 5′-AGATGTGATGGTATGAGGCAAGT-3′ |  |  |  |
| **3** | 5' Untranslated Region (UTR ) and Exon 1 | F: 5′-AAGAACCTGCTCATGTAACCAAA-3′ | 1169 | 64 | 30 |
|  |  | R: 5′-GACTTGTGCTAGCTGTTCTCTCC-3′ |  |  |  |
| **4** | Intron 1 | F: 5′-GGGCTATGTTTATTGTGCAGTTC-3′ | 1177 | 64 | 35 |
|  |  | R: 5′-ATCCTGGTCTGCTTCTAGATTCC-3′ |  |  |  |
| **5** | Intron 1 | F: 5′-ATCTCCAACCATCTGACATTCC-3′ | 1095 | 64 | 31 |
|  |  | R: 5′-CATCTTGTCTTCCCTTCATTCC-3′ |  |  |  |
| **6** | Intron 1 | F: 5′-AGGAGTTTGGGTCTTATTTAGGC-3′ | 903 | 64 | 35 |
|  |  | R: 5′-CATGATCTTAGCACCTAGCCTTG-3′ |  |  |  |
| **7** | Intron 1 | F: 5′-GAGGGAAGGTGAGCTAATCTGTT-3′ | 2002 | 64 | 32 |
|  |  | R: 5′-CACAAAGAGTTTGAACCGACTAG-3′ |  |  |  |
| **8** | Intron 1 | F: 5′-CTCAGGATCACCTGGATTCCTAT-3′ | 1181 | 64 | 35 |
|  |  | R: 5′-GTTCAAGCCACTGCACTCTACTC-3′ |  |  |  |
| **9** | Intron 1 | F: 5′-TAGGAGAGGCTTGTGTTTGTTTC-3′ | 1182 | 64 | 33 |
|  |  | R: 5′-AAAGGTTAGGAGGACATCAGAGG-3′ |  |  |  |
| **10** | Exon 2 | F: 5′-AATATCTGCCTTCTCCAAAAACC-3′ | 1135 | 64 | 34 |
|  |  | R: 5′AGCCCACTAACTGTACAATCTGC-3′ |  |  |  |
| **11** | Intron 2 | F: 5′-GTTGGAATTAACTGCAGAAGCTG-3′ | 1199 | 64.6 | 35 |
|  |  | R: 5′-GCCCTTTGTGAAAACATAGGAAC-3′ |  |  |  |
| **12** | Intron 2 and Exon 3 | F: 5′-CTGAGCATCCCTGGTTAGAAGA-3′ | 1110 | 64.6 | 35 |
|  |  | R: 5′-ACAAAAAGAGAGGAGGCCATTC-3′ |  |  |  |
| **13** | Exon 3 | F: 5′-ATGGCTGGCTACACCCTAAgta-3′ | 1169 | 64 | 35 |
|  |  | R: 5′-TCAGCTGGAATATTTGTCAGGA-3′ |  |  |  |
| **14** | Intron 3, Exon 4 and Intron 4 | F: 5′-GCAGGACTCTACCTCCTATGCTT-3′ | 1947 | 53 | 30 |
|  |  | R: 5′-ACACCTTGTTTGGACTGATTCTG-3′ |  |  |  |
| **15** | Exon 5 | F: 5′-ATAGAGCAGGCATTTGATCTTGA-3′ | 1135 | 64 | 36 |
|  |  | R: 5′-CCCCTTCCTAGATTTCACCTTTA-3′ |  |  |  |
| **16** | Exon 5 and Exon 6 | F: 5′-GAGGAAAATGCCAGATAGTGATG-3′ | 1196 | 55.9 | 30 |
|  |  | R: 5′-GCCAGGCACTGACTTACTACAAA-3′ |  |  |  |
| **17** | Intron 6 | F: 5′-GGCACACTTGTTTGCTCTTCTAT-3′ | 1148 | 51 | 37 |
|  |  | R: 5′-CGTGTAACCTTGATTTTCCTGTC-3′ |  |  |  |
| **18** | Intron 6 | F: 5′-GTGTTGGGAGGATTAAGGAGATT-3′ | 1135 | 55.9 | 30 |
|  |  | R: 5′-ATGGCTACTGTTCACTGCAGATT-3′ |  |  |  |
| **19** | Exon 7 | F: 5′-CTCAAGGTGACACAGCTCATAAA-3′ | 1045 | 64 | 38 |
|  |  | R: 5′-TGGGAGATGGAGTTGTACTCTGT-3′ |  |  |  |
| **20** | Intron 7 | F: 5′-CACAGTACTTTGAGAAGCCAAGG-3′ | 1175 | 55.9 | 30 |
|  |  | R: 5′-TCCTGGGAGGAGTATAAAATGGT-3′ |  |  |  |
| **21** | Intron 7 | F: 5′-TGTAAGGTCTCCCTTTTCTCTTC-3′ | 1078 | 64 | 39 |
|  |  | R: 5′-TGTAAGGTCTCCCTTTTCTCTTC-3′ |  |  |  |
| **22** | Intron 7 | F: 5′-CTCGTCCCTCTGTCTTAGTCCTT-3′ | 1005 | 64 | 35 |
|  |  | R: 5′-TGATCAAAGAATTTGGCTTCATT-3′ |  |  |  |
| **23** | Exon 8 | F: 5′-TAAATAGCCAGTATCTGGGGACA-3′ | 1112 | 64 | 40 |
|  |  | R: 5′-TTGACCCTCTCTTGATGCTTAAC-3′ |  |  |  |
| **24** | Intron 8 | F: 5′-GGCATTGTTAAGAAATCACGTTC-3′ | 1058 | 61 | 35 |
|  |  | R: 5′-TGATCCAGTAATTCCACTTTTGG-3′ |  |  |  |
| **25** | Intron 8 | F: 5′-CTCCACTTCTTGGCTATTGTGAA-3′ | 1138 | 64 | 41 |
|  |  | R: 5′-AAGGCCTGTACCCAAATGTCTAT-3′ |  |  |  |
| **26** | Intron 8 | F: 5′-AGTGTGCCACAATTCATACACTG-3′ | 1146 | 56.7 | 35 |
|  |  | R: 5′-GTTTGGCCTGGCTATTTTAGACT-3′ |  |  |  |
| **27** | Intron 8 | F: 5′-ACTGGGACTGAGTACAGGAGATG-3′ | 1061 | 64 | 42 |
|  |  | R: 5′-AAGTCACGCTGTCTACTTTGGAA-3′ |  |  |  |
| **28** | Intron 8 | F: 5′-TGCCTAGTGTGTGAGGTCTAAGG-3′ | 1046 | 61 | 35 |
|  |  | R: 5′-CTACCATGCCCAGTTAATTTTTG-3′ |  |  |  |
| **29** | Intron 8 | F: 5′-TGAACACAATGCAAAGGAGAATA-3′ | 1086 | 64 | 43 |
|  |  | R: 5′-GCTTGGTTGGATAAAATTCACTG-3′ |  |  |  |
| **30** | Intron 8 | F: 5′-TGTTCTCGAACTCCTGACCTTAG-3′ | 1034 | 56.7 | 35 |
|  |  | R: 5′-TCCCTAGTATGCTTTTGTGCATT-3′ |  |  |  |
| **31** | Intron 8 | F: 5′-AGACGAAACATTTCCGACATTTA-3′ | 1085 | 64 | 44 |
|  |  | R: 5′-CTACCATCTGTGCCATTTCTAGG-3′ |  |  |  |
| **32** | Intron 8 | F: 5′-GAATACAAAATCGAGCATGGAAC-3′ | 1002 | 56.7 | 35 |
|  |  | R: 5′-TTTTTGCTGGGTCAAGAATTTTA-3′ |  |  |  |
| **33** | Intron 8 | F: 5′-GACCCTGTCTCAAAACAAAAACA-3′ | 823 | 64 | 45 |
|  |  | R: 5′-GCTAACATGGTGAAACCCTTTCT-3′ |  |  |  |
| **34** | Intron 8 | F: 5′-TCTTGGCTTACTGCAAGCTCTAC-3′ | 1032 | 56.7 | 35 |
|  |  | R: 5′-GTCTCCACTAGAGAACCCCTGAT-3′ |  |  |  |
| **35** | Intron 8 | F: 5′-ATCTACCCTTCCCTATGTTTCCA-3′ | 932 | 64 | 46 |
|  |  | R: 5′-CTCAGCACCTCAGAAGAAAACTG-3′ |  |  |  |
| **36** | Intron 8 | F: 5′-TCATACCCACTTTCACTCTAGCC-3′ | 1050 | 56.7 | 35 |
|  |  | R: 5′-CATCACTCTTTTCTCCCTCCTTT-3′ |  |  |  |
| **37** | Exon 10 | F: 5′-GGCACAGGTGAATACAAAAAGAG-3′ | 1098 | 64 | 47 |
|  |  | R: 5′-AATCCACAGAACTGAGCCTGTAA-3′ |  |  |  |
| **38** | Intron 10 | F: 5′-CAATCTTCTGTCCTCTGAGCAAT-3′ | 1922 | 61 | 30 |
|  |  | R: 5′-TTCGAGCTTATAGCCTGTTTTTG-3′ |  |  |  |
| **39** | Intron 10, Exon 11 and 3’ UTR | F: 5′-GAAGGAATGGTTGTGTCTGGATA-3′ | 1165 | 55 | 30 |
|  |  | R: 5′-CTTACTTGAGCCCAGAAATTCAA-3′ |  |  |  |
| **40** | 3' Downstream | F: 5′-CAACAGTGAGAGCCTGTGTCTAC-3′ | 904 | 64 | 49 |
|  |  | R: 5′-CCCATTAGAAGCTTTTTCCTGAT-3′ |  |  |  |
